# Identification of potential NUDT5 inhibitors from marine bacterial natural compounds via molecular dynamics and free energy landscape analysis

**DOI:** 10.1007/s11030-024-10950-5

**Published:** 2024-09-03

**Authors:** Amit Dubey, Amer M. Alanazi, Rima Bhardwaj, Andrea Ragusa

**Affiliations:** 1 Department of Pharmacology, Saveetha Dental College, and Hospitals, Saveetha Institute of Medical and Technical Sciences, Saveetha University, Chennai, Tamil Nādu 600077 India; 2 Department of Computational Chemistry and Drug Discovery Division, Quanta Calculus, Greater Noida, 201310 India; 3 Pharmaceutical Biotechnology Laboratory, Department of Pharmaceutical Chemistry, College of Pharmacy, King Saud University, Riyadh, Saudi Arabia; 4 Department of Chemistry, Poona College, Savitribai Phule Pune University, Pune, India; 5 Institute of Nanotechnology, CNR-Nanotec, Via Monteroni, 73100 Lecce, Italy; 6 Department of Life Sciences, Health and Health Professions, Link Campus University, Via del Casale Di San Pio V 44, 00165 Rome, Italy

**Keywords:** NUDT5, Marine bacterial compounds, Breast cancer, Molecular docking, Molecular dynamics, Free energy landscape

## Abstract

NUDIX hydrolase 5 (NUDT5) is an enzyme involved in the hydrolysis of nucleoside diphosphates linked to other moieties, such as ADP-ribose. This cofactor is vital in redox reactions and is essential for the activity of sirtuins and poly(ADP-ribose) polymerases, which are involved in DNA repair and genomic stability. It has been shown that NUDT5 activity can also influence NAD+ homeostasis, thereby affecting cancer cell metabolism and survival. In this regard, the discovery of NUDT5 inhibitors has emerged as a potential therapeutic approach in cancer treatment. In this study, we conducted a high-throughput virtual screening of marine bacterial compounds against the NUDT5 enzyme and four molecules were selected based on their docking scores. These compounds established strong interactions within the NUDT5 active site, with molecular analysis highlighting the key role of Trp^28A^ and Trp^46B^ residues. Molecular dynamics simulations over 200 ns indicated a stable behavior, in association with root mean square deviation values always below 3 Å, suggesting conformational stability. Free energy landscape analysis further supported their potential as NUDT5 inhibitors, offering avenues for novel therapeutic strategies against NUDT5-associated breast cancer.

**Supplementary Information:**

The online version contains supplementary material available at 10.1007/s11030-024-10950-5.

## Introduction

Breast cancer remains a leading cause of cancer-related deaths, particularly among women, due to its complex nature, metastatic potential, and resistance to treatments [1]. Despite advancements in medical research, it ranks as the second deadliest cancer in females, with millions of new cases and hundreds of thousands of deaths recorded annually. The classification of breast cancer into molecular subtypes adds to its complexity.

While there has been improvement in the 5-year survival rates across all subtypes and stages, metastatic breast cancer still presents significant challenges, with lower survival rates. The World Health Organization aims to substantially reduce breast cancer mortality by 2030–2040, emphasizing the importance of early detection, timely diagnosis, and comprehensive disease management strategies [2, 3]. Despite progress in genomic and proteomic methods, treatment failure in breast cancer persists, largely due to its heterogeneous nature and the development of drug resistance. Understanding the molecular mechanisms underlying breast cancer, including genetic mutations, tumor microenvironment, and cellular signaling pathways, is crucial for improving treatment outcomes.

NUDIX hydrolases constitute a diverse family of enzymes involved in cellular metabolism, particularly in the hydrolysis of nucleoside diphosphate derivatives [4]. Among them, NUDT5 (Nucleoside Diphosphate-linked moiety X-type motif 5 or NUDIX 5) has garnered increasing attention due to its role in regulating several cellular processes, including modulation of calcium signaling and of ADP-ribose levels [5]. This enzyme thus plays a significant role in cellular metabolism, particularly in the regulation of NAD+, a vital cofactor in redox reactions and essential for the activity of sirtuins and poly(ADP-ribose) polymerases (PARPs), which are involved in DNA repair and genomic stability. Cancer cells often exhibit altered NAD+ metabolism to support their rapid proliferation and survival under stress conditions and it has been shown that modulation of the NUDT5 activity influences NAD+ homeostasis, thereby affecting cancer cell metabolism and survival [6–8]. Given its role in maintaining NAD+ levels, NUDT5 has emerged as a potential therapeutic target in cancer treatment and there is considerable interest in identifying inhibitors of NUDT5. Several studies already identified small molecules that can effectively inhibit NUDT5 activity, thereby offering potential therapeutic benefits [6, 9–11]. However, the search for more effective and specific inhibitors with fewer side effects continues.

Effective drug discovery is essential for developing novel cancer therapies and screening of natural products already led to promising results [12, 13]. Marine algae and marine bacteria have been found to produce a wide array of secondary metabolites with unique structures and potent biological activities, thus representing a rich source of bioactive molecules with diverse chemical compositions [14, 15]. These natural products have proven to be valuable sources of new drugs and lead compounds in various therapeutic areas [16, 17]. Nevertheless, traditional screening methods are often costly and time-consuming, prompting researchers to explore computational approaches. In this regard, computational tools offer a streamlined process for identifying potential drug candidates, from the high-throughput virtual screening (HTVS) of large libraries, to molecular docking of selected compounds and molecular dynamics validation of the results [18].

In this study, we identified and characterized potential NUDT5 inhibitors from a library of marine bacterial natural compounds. To that aim, we employed a multi-step computational approach that included high-throughput virtual screening, molecular docking, molecular dynamics (MD) simulations, and binding free energy calculations. High-throughput virtual screening allowed for the rapid assessment of large compound libraries to identify those with the highest potential for binding to the target protein [19]. Molecular docking further refined this selection by predicting the preferred orientation of the compounds within the binding site of the target protein [20]. MD simulations were used to evaluate the stability and dynamics of the protein-ligand complexes in a simulated physiological environment, providing insights into the conformational changes and interactions that occur over time [21]. Finally, binding free energy calculations, using the Molecular Mechanics Poisson-Boltzmann Surface Area (MM/PBSA) method, offered quantitative estimates of the binding scores, contributing to the selection of the most promising compound [22].

## Methodology

### Protein structure collection and preparation

The crystal structure of the NUDT5 protein was collected from the RCSB Protein Data Bank (PDB), having the PDB ID 5NQR [5, 23]. 5NQR was selected due to its high-resolution structure and relevance in previous studies related to NUDT5 inhibitors [5]. Alternative structures, such as PDB ID 5NWH, were also considered, but 5NQR had better resolution and thus provided a better template for docking studies. The 5NQR protein structure was obtained by X-ray diffraction technique, the resolution of the structure was 2.20 Å, and the molecular weight was 49.9 kDa. Although the protein is a homodimer, both chains have been retained in the simulations because the active site is located at the interface between the two chains. Unwanted ions and water molecules were eliminated from the crystal structure during protein preparation. Hydrogen atoms and missing loops were added and charges were incorporated into the protein structure to balance the protonation state at pH 7.0. The protein preparation steps were performed using the UCSF Chimera software, version 1.17.3 [24]. The prepared structure was energy minimized and utilized for the subsequent computational screening and validation process.

### Ligand library selection

Marine bacterial compounds were selected for virtual screening due to their unique and diverse chemical structures. The 2895 natural compounds were downloaded as SDF files from the Comprehensive Marine Natural Products Database (CMNPD) by applying the “Bacteria” filter in the “Taxonomy” subgroup and collected into a ligand library for the subsequent virtual screening analysis [25]. Compounds with bad valencies and those lacking drug-like properties were removed from the library and the remaining natural compounds were selected as ligands to be screened against the NUDT5 protein [26].

### Virtual screening and re-docking analyses

Virtual screening is a computational approach used in drug discovery and other fields to efficiently scan large databases of molecules for potential candidates with desired properties. A high-throughput virtual screening analysis of the selected natural compounds against the prepared NUDT5 protein was carried out using the MTiOpenScreen web server, thus obtaining a ranking of the ligands based on their binding score [27]. A computer grid was constructed around the active site of the target protein, as defined by the position of the co-crystallized native ligand (compound 958) in the 5NQR protein structure. The grid measured 20 × 20 × 20 Å in dimensions and was centered at the following coordinates: X: 77.3 Å, Y: 16.36 Å, and Z: 111.16 Å.

To validate the binding strength and study the specific interactions within the docked complex, the compounds with binding scores lower than − 10.5 kcal/mol were re-docked within the binding site under identical conditions as the initial virtual screening by using the AutoDock Vina plugin in the Chimera interface [28]. For comparison and validation purposes, the native ligand 958 was also re-docked alongside the selected compounds. Visualizations of the 3D structures of the docked complexes were created using Maestro visualization software, while 2D interaction diagrams were generated through Maestro’s ligand interaction module (Schrödinger Release 2020-4: Maestro, Schrödinger, LLC, New York, NY, 2020).

### Molecular dynamics simulations

MD simulations were performed to evaluate the time-dependent behavior and the conformational changes of the complexes, providing valuable insights into their structure, dynamics, and thermodynamics. The CHARMM36 force field, chosen for its accuracy in simulating protein–ligand interactions and its widespread use in similar studies, was utilized in the GROMACS simulation platform for a 200 ns long analysis of the NUDT5 protein with each of the four natural compounds as well as the native ligand [29, 30]. Ligand preparation involved positioning each docked complex centrally in a cubic simulation box, with TIP3P water surrounding each complex and maintaining a minimum buffer distance of 1 nm from the protein to the box boundary. Sodium and chloride ions were added to neutralize the system, achieving an ionic strength equivalent to a 0.15 M concentration. A steepest minimization phase was conducted to eliminate steric hindrance and geometric divergence until the maximal force fell below 1000 kJ/mol/nm, preparing the systems for subsequent MD simulations.

The systems were first balanced at 310 K using a V-rescale thermostat for the NVT phase, and then achieved pressure stabilization at 1 bar with the Parrinello-Rahman barostat for the NPT phase. Both phases lasted 100 ps to establish stable temperature and pressure conditions. The Particle Mesh Ewald approximation was used to handle electrostatic, Coulomb, and van der Waals interactions. Subsequently, MD simulations capturing the dynamic behavior of the complexes were conducted over a final 200 ns production run employing the CHARMM36 force field. Temperature and pressure control consistent with the equilibration phases were maintained, while periodic boundary conditions simulated an infinite environment. Trajectories were assessed through several metrics, such as Root Mean Square Deviation for global deviation, Root Mean Square Fluctuation for residue flexibility, and analysis of hydrogen bonding patterns, for evaluating the robustness and stability of the interactions by determining the number and duration of hydrogen bonds between the target protein and the inhibitors.

### Principal component and free energy landscape analyses

The Principal Component Analysis (PCA) on simulated protein–ligand trajectories was utilized to identify key dynamic interactions. The GROMACS’ 'gmx sham' module was employed to focus on the essential movements of NUDT5 protein’s core atoms and to highlight their influence on ligand binding. The resulting principal components (PC1, PC2, and PC3) were derived using the ‘gmx anaeig’ module, while ‘gmx anaproj’ was utilized for visualizing and reducing the multidimensional dataset [31]. The principal components were used to create Free Energy Landscape (FEL) analyses, visualized using Geo-measure, a pymol plugin [32, 33]. The landscapes illustrated the distribution of structural states and stable conformations as low-energy troughs, while 3D plots were utilized to analyze complex flexibility and stability, revealing both stable conformations and transition states. This provided insights into inhibitor binding scores and specificities, identifying energetically efficient binding patterns and estimating inhibitor efficiency. PCA and FEL analyses were generated using the trajectories from the last 100 ns of the MD simulations to ensure that the system had reached equilibrium. The commands used for these analyses in GROMACS were as follows:

gmx covar -s topol.tpr -f traj.xtc -o eigenval.xvg -v eigenvec.trr -av average.pdb;

gmx anaeig -v eigenvec.trr -f traj.xtc -s topol.tpr -2d PCA.xvg;

gmx sham -f PCA.xvg -ls free_energy_landscape.xvg.

### Binding free energy calculation using MM/PBSA method

The MM/PBSA method was exploited through the GMXPBSA tool for calculating the free binding energy of all the docked complexes [34, 35]. This computational tool included PCM and SGB models for solvation energy calculations, along with consideration of van der Waals and electrostatic interaction energies to evaluate overall energy within each complex.

## Results

### Virtual screening and re-docking analysis

The active site of the NUDT5 protein was screened against 2895 natural compounds with drug-like properties derived from marine bacteria and selected from the CMNPD database (Table S1) [25]. Based on the virtual screening results, the docked compounds showed binding scores ranging from − 11.2 to − 4.0 kcal/mol. The compounds with binding scores equal or lower than − 10.5 kcal/mol were selected for the validation process, i.e., the compounds with IDs CMNPD20698, CMNPD24402, CMNPD20696, and CMNPD19658, exhibiting binding scores of − 11.2, − 10.6, − 10.6, and − 10.5 kcal/mol, respectively. These compounds were docked again with higher accuracy with the target protein in the same binding pocket of the co-crystallized native ligand 958 for fine-tuning and validating the binding scores. The native ligand 958 was also re-docked in the same position during this comparative study. Interestingly, the NUDT5-CMNPD20698 complex exhibited the highest negative re-docking score (− 11.1 kcal/mol), followed by the NUDT5-CMNPD20696, the NUDT5-CMNPD24402, and the NUDT5-CMNPD19658 complexes (re-docking scores of − 10.8, − 10.6, and − 10.5 kcal/mol, respectively, Table 1). These docking scores suggest strong affinities, comparable to those of known NUDT5 inhibitors.

**Table 1:** List of the selected natural compounds and of the reference ligand with their respective chemical structure, binding energy, and re-docking score

S.no	Compound ID	Compound Name	Compound Structure	Binding energy (kcal/mol)	Re-docking score (kcal/mol)
1	CMNPD20698	Marinacarboline D		− 11.2	− 11.1
2	CMNPD24402	Metagenetriindole A		− 10.6	− 10.6
3	CMNPD20696	Marinacarboline B		− 10.6	− 10.8
4	CMNPD19658	Dermacozine C		− 10.5	− 10.5
5	958 (native ligand)	8-(dimethylamino)-1,3-dimethyl-7-[[5-(3-methylphenyl)-1,3,4-oxadiazol-2-yl]methyl]purine-2,6-dione		-	− 9.0

Noteworthy, the re-docking score of the NUDT5-958 reference complex (− 9.0 kcal/mol) was higher than those of the re-docked complexes, suggesting that the selected natural compounds should bind the enzyme even more strongly than the native ligand and were thus considered for a detailed validation analysis.

### Intermolecular interaction analysis

**Fig. 1:**
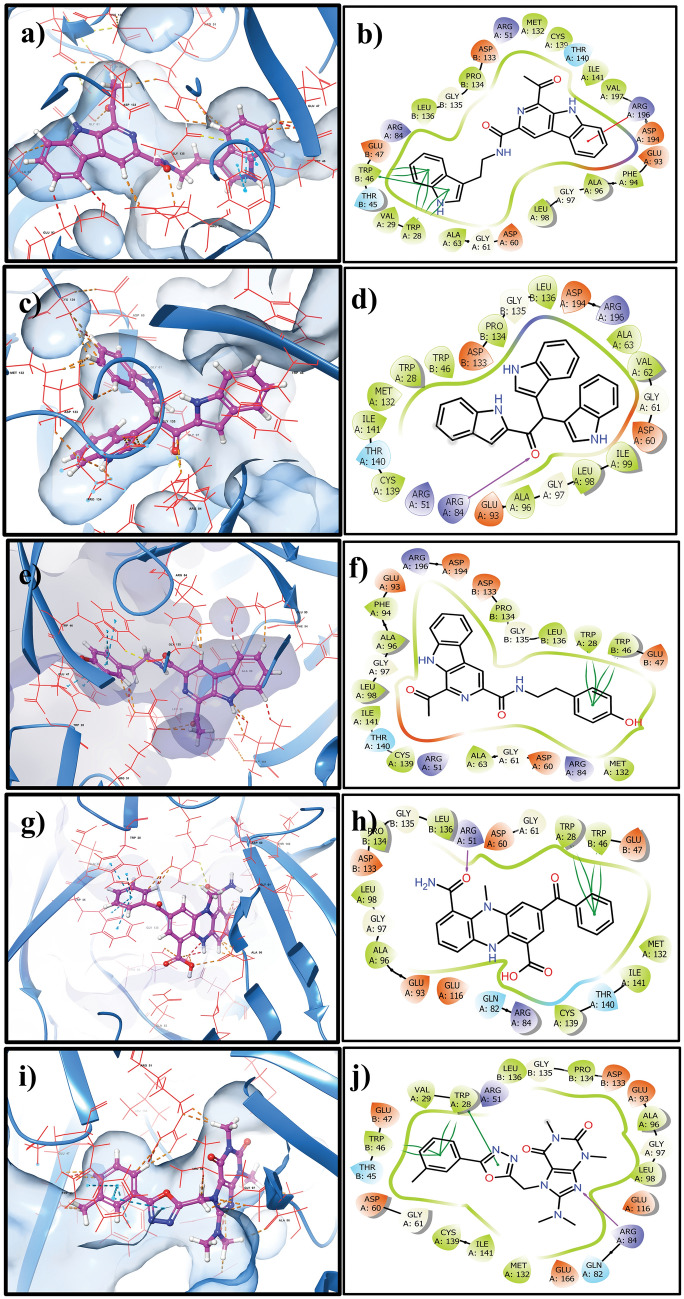
3D (left) and 2D (right) images of the interactions between the NUDT5 protein and the selected natural compounds: **a**, **b** CMNPD20698, **c**, **d** CMNPD24402, **e**, **f** CMNPD20696, and (**g**, **h**) CMNPD19658, and reference compound **i**, **j** 958

The intermolecular interactions between the protein and ligand define the stability of each complex. As reported in Table 2 and Fig. 1, various intermolecular interactions were formed between the protein and the marine bacteria compounds. In particular, the NUDT5-CMNPD20698 complex exhibited π–π stacking interactions with the Trp^28A^ and Trp^46B^ residues and established a π–cation interaction with the Arg^196A^ residue present in the binding site of the receptor protein. In the case of NUDT5-CMNPD24402 complex, formation of a single hydrogen bond with the Arg^84A^ residue was noted. However, π–π stacking bonds were observed again with Trp^28A^ and Trp^46B^ in the NUDT5-CMNPD20696 complex, similarly to NUDT5-CMNPD20698. Interestingly, both hydrogen bond and π–π stacking interactions were also present in the NUDT5-CMNPD19658 complex with the Arg^51A^ and the Trp^28A^ and Trp^46B^ residues, respectively. The reference complex showed a similar pattern of interactions, although this time it was a different arginine, i.e., Arg^84A^, to participate to the hydrogen bond, while Trp^28A^ and Trp^46B^ contributed again to the π–π stacking. These two residues in particular consistently formed strong π–π stacking with the all the studied compounds, contributing to their binding affinity and stability, thus evidencing their importance within the active site. Other than these two significant interactions, hydrophobic contacts were also formed with several residues within the active site, contributing to maximize the binding affinity of each complex.

**Table 2:** List of interaction types and corresponding protein residues interacting with the selected natural compounds and the reference ligand in each complex

S.no	Complex	H-bond	Hydrophobic	π-π stacking	π-cation
1	NUDT5-CMNPD20698	–	Trp^28A^, Val^29A^, Ala^63A^, Phe^94A^, Ala^96A^, Leu^98A^, Met^132A^, Cys^139A^, Ile^141A^, Val^197A^, Trp^46B^, Pro^134B^, Leu^136B^	Trp^28A^, Trp^46B^	Arg^196A^
2	NUDT5-CMNPD24402	Arg^84A^	Ala^63A^, Val^62A^, Ala^96A^, Leu^98A^, Ile^99A^ Met^132A^, Cys^139A^, Ile^141A^, Trp^46B^, Pro^134B^, Leu^136B^	–	–
3	NUDT5-CMNPD20696	-	Trp^28A^, Ala^63A^, Phe^94A^, Ala^96A^, Leu^98A^, Met^132A^, Cys^139A^, Ile^141A^, Trp^46B^, Pro^134B^, Leu^136B^	Trp^28A^, Trp^46B^	–
4	NUDT5-CMNPD19658	Arg^51A^	Trp^28A^, Ala^96A^, Leu^98A^, Met^132A^, Cys^139A^, Ile^141A^, Trp^46B^, Pro^134B^, Leu^136B^	Trp^28A^, Trp^46B^	–
5	NUDT5-958	Arg^84A^	Trp^28A^, Val^29A^ Ala^96A^ Leu^98A^, Met^132A^, Cys^139A^, Ile^141A^, Trp^46B^, Pro^134B^, Leu^136B^	Trp^28A^, Trp^46B^	–

### Molecular dynamics simulation analysis

The docked complexes were analyzed using a 200 ns long MD simulation to examine their conformational stability and hydrogen bond patterns over time. The study focused on understanding the interactions and movements within the complexes by closely examining key structural indicators. Analyzing the Root Mean Square Deviation (RMSD) during the entire simulation period was crucial for evaluating structural consistency, changes over time, and assessing the stability of the protein–ligand binding interactions. To further study the protein structure, the Root Mean Square Fluctuation (RMSF) analysis was also used. This approach shows conformational shifts and flexibility in individual amino acid residues during the simulation. It identified regions with structural changes or increased mobility, giving insights into complex dynamics. We also looked at the formation of hydrogen bonds within each complex over the 200 ns simulation period to understand their stability and importance within the protein-ligand complexes.

#### RMSD and RMSF analyses

**Fig. 2:**
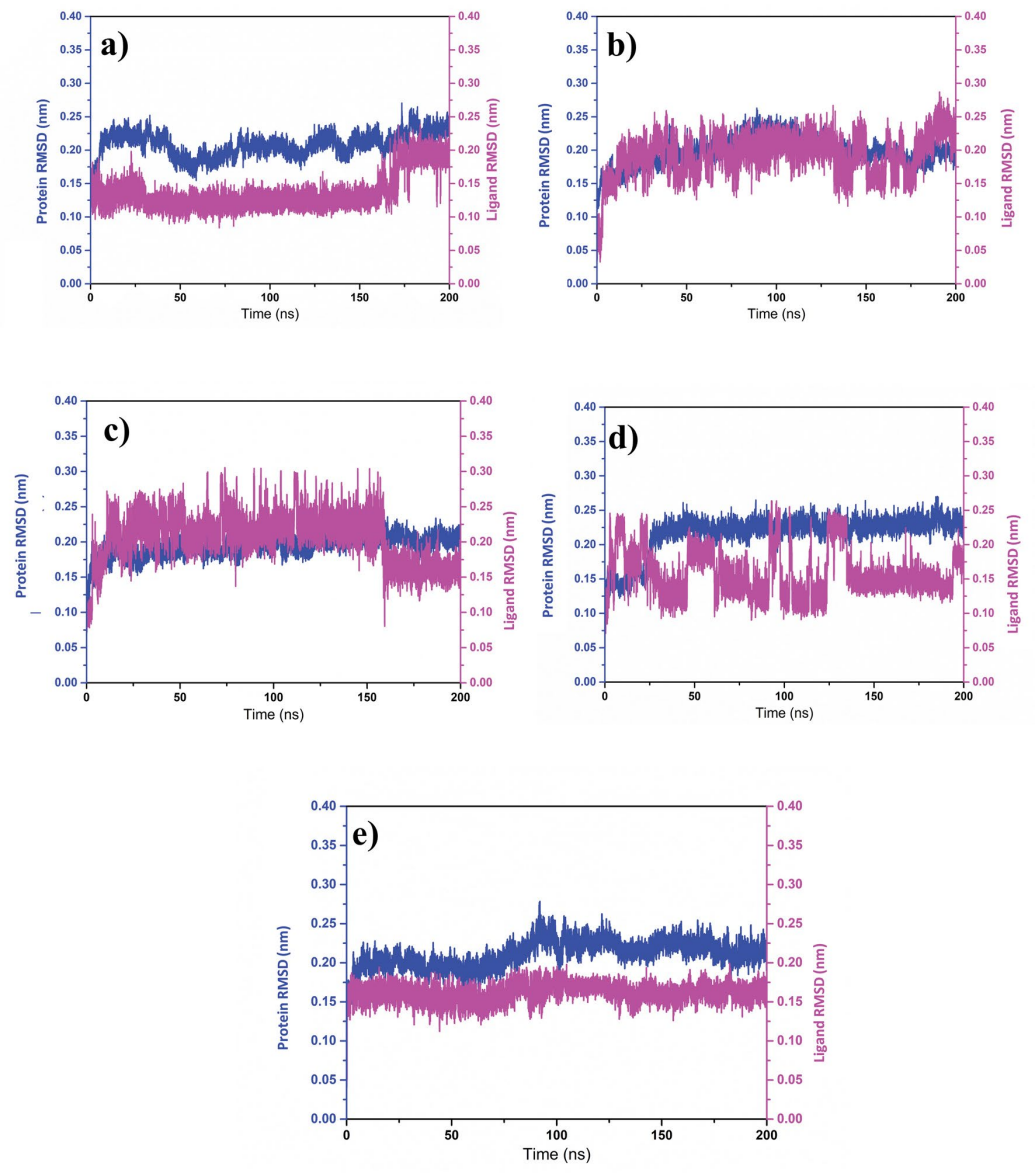
RMSD plots retrieved from the MD simulation trajectory of the NUDT5 protein and the selected natural compounds: **a** CMNPD20698, **b** CMNPD24402, **c** CMNPD20696, **d** CMNPD19658, and the reference compound **e** 958

MD simulations revealed the formation of stable protein-ligand complexes, with RMSD values always within 1–3 Å. The RMSD values for the selected compounds also remained stable throughout the 200 ns simulation period, indicating robust binding (Fig. 2). In the NUDT5-CMNPD20698 complex, the protein RMSD was always 0.25 nm, while the ligand RMSD was around 0.15 nm till about 160 ns and, by the end of the simulation, the value was 0.20 nm, confirming that the protein-ligand complex was in a stable state throughout the simulation. Similarly, in NUDT5-CMNPD24402 complex, the protein RMSD was 0.20 nm and the ligand RMSD was 0.25 nm. In the case of the NUDT5-CMNPD20696 complex, a similar protein RMSD around 0.20 nm was observed, but the ligand RMSD on average was < 0.25 nm till about 150 ns, but this value was later reduced to less than 0.20 nm till the end of the simulation. Furthermore, in the NUDT5-CMNPD19658 complex, the protein RMSD was always less than 0.25 nm and the ligand RMSD showed only minor fluctuations, with a maximum RMSD of 0.25 nm after about 90 and 130 ns, but average values always below 0.20 nm. Also, the RMSD plot analysis of the reference complex NUDT5-958 showed a protein RMSD value < 0.25 nm and a ligand RMSD value < 0.20 nm.

Altogether, the RMSD plot analysis of the selected ligands and of the reference compound bound to the protein showed that the binding of any of the molecules did not induce a significant conformational change in the enzyme. On the other hand, the binding of the ligands provided stability to the protein structure, similar to the reference ligand, suggesting that the selected natural compounds may have the potential to inhibit the functionality of the target protein.

**Fig. 3:**
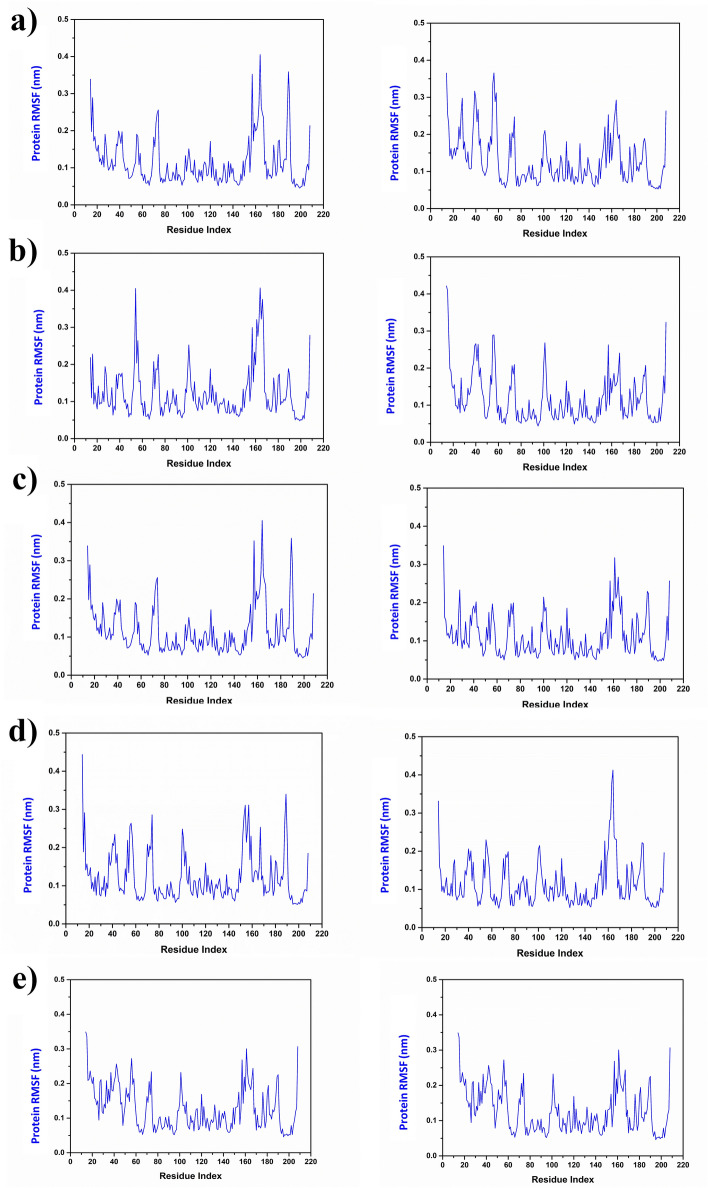
Protein RMSF plots of chain A (left) and chain B (right) of the NUDT5 protein docked with compounds **a** CMNPD20698, **b** CMNPD24402, **c** CMNPD20696, **d** CMNPD19658, and **e** the reference compound 958

Furthermore, the flexibility of NUDT5 and the selected natural compounds in the respective complexes against the NUDT5-958 inhibitor complex was monitored via RMSF analysis of the respective 200 ns MD simulation trajectories. Notably, residues of both chains in the target protein in all complexes docked with the selected natural compounds demonstrated similar RMSF values < 0.4 nm. Interestingly, residues between 140 and 200 in both chains showed fluctuations during the simulation, but these fluctuations did not contribute to significant conformational or structural changes in the target protein. Similar fluctuations were observed in the reference complex, further confirming that the binding of the natural compounds did not affect the protein structure during the simulation (Fig. 3).

#### Hydrogen bond analysis

**Fig. 4:**
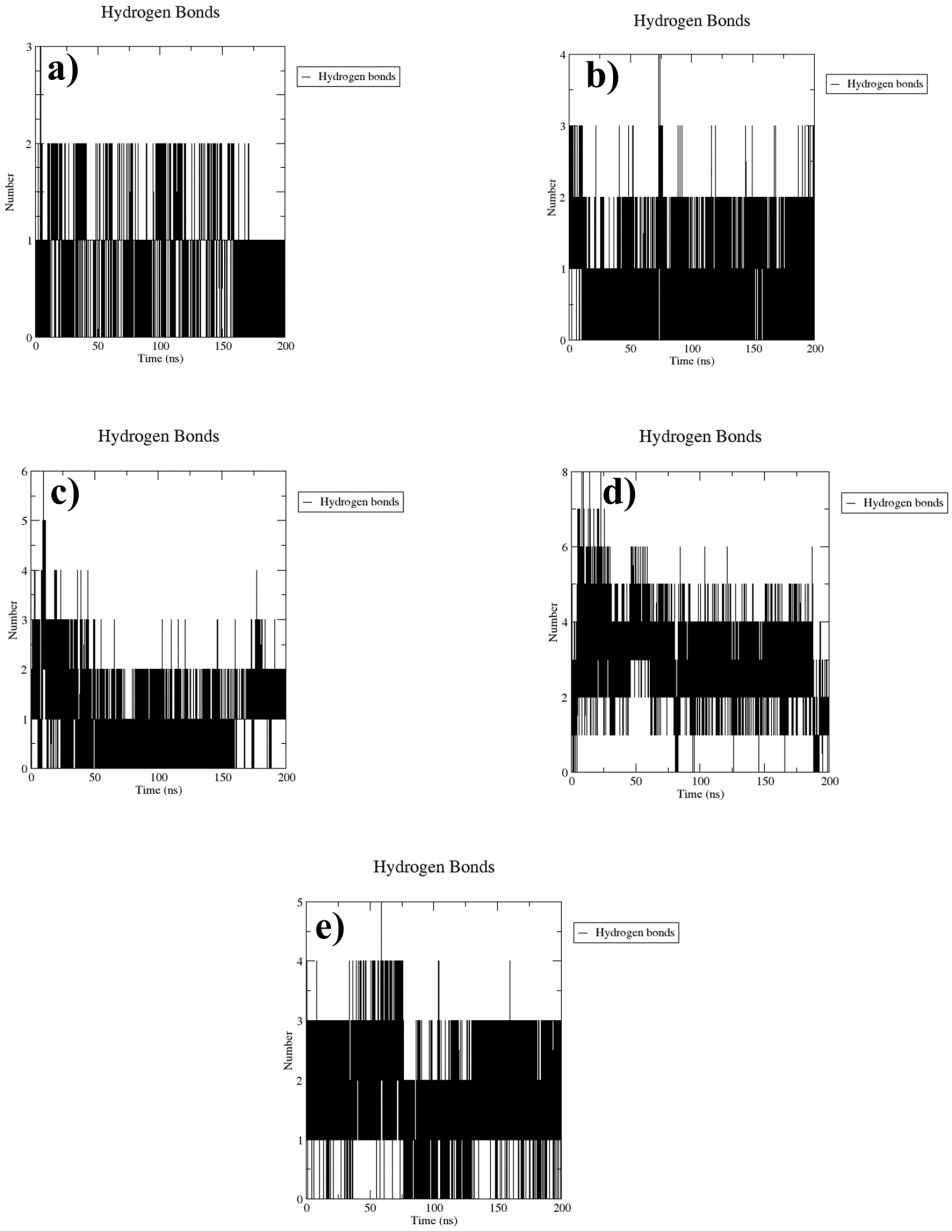
Number of hydrogen bonds formed over time during the MD simulations of the NUDT5 protein docked with the natural compounds **a** CMNPD20698, **b** CMNPD24402, **c** CMNPD20696, **d** CMNPD19658, and the reference compound **e** 958

It is essential to comprehend the molecular interactions responsible for creating hydrogen bonds, as this has a significant impact on revealing the strength and stability of binding in the complexes. During the MD simulation, the NUDT5-CMNPD20698 complex consistently formed two hydrogen bonds till 180 ns but, by the end of the simulation, only one hydrogen bond was present. The NUDT5-CMNPD24402 complex also exhibited the formation of two to three hydrogen bonds during the simulation period. The NUDT5-CMNPD20696 complex formed three hydrogen bonds by the end of the simulation, with an additional display of four to five hydrogen bonds within the initial 20 ns. The NUDT5-CMNPD19658 complex demonstrated the formation of the highest number of hydrogen bonds, which was around six to eight for the first 20 to 60 ns, and around four to five bonds throughout the whole simulation period. In contrast, the reference complex displayed three hydrogen bonds most of the simulation time, but four hydrogen bonds were formed around 40 to 60 ns. When comparing all these results, except NUDT5-CMNPD20698, all other complexes exhibited the formation of a significant number of hydrogen bonds, supporting the fact that the selected natural compounds possess a strong binding affinity that contributes to better dynamic stability of the formed complexes (Fig. 4).

### Principal component analysis

**Fig. 5:**
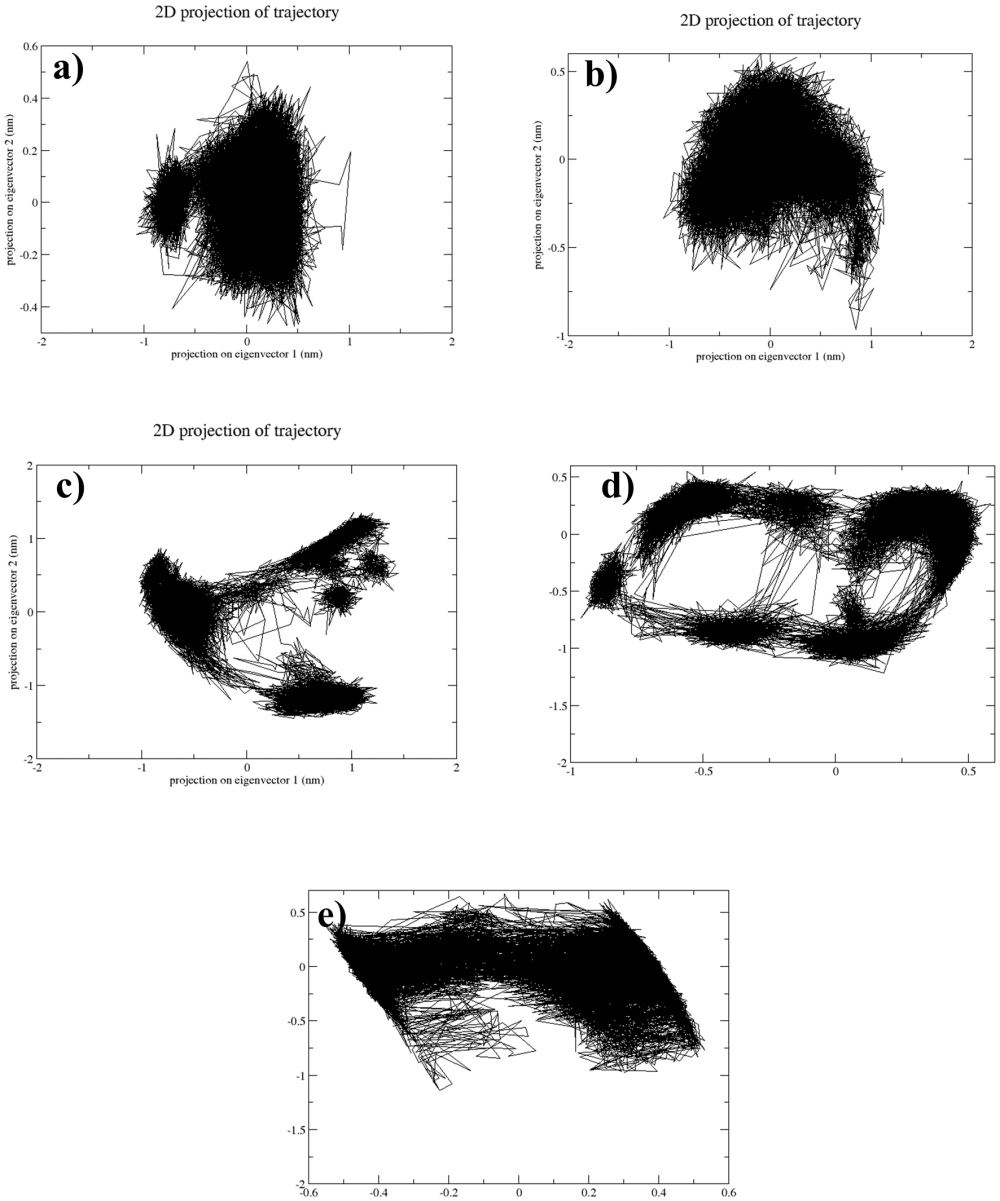
PCA scatter plots of PC1 vs PC2 of the NUDT5 protein docked with the natural compounds **a** CMNPD20698, **b** CMNPD24402, **c** CMNPD20696, **d** CMNPD19658, and the reference compound **e** 958

Macromolecules exhibit functional movements due to correlations among individual atoms, crucial for proper functioning and adaptation. Understanding these internal motions in proteins is challenging, but PCA simplifies high-dimensional datasets by identifying major components, and this approach has already been successfully exploited to investigate binding interactions [36, 37]. The variance from the data is represented as principal components (e.g., PC1 and PC2), and in this case, the PCA analysis gives insight into the significant conformational changes in the protein-ligand complex by clustering similar conformations of the protein-ligand complex. In the scatter plots of NUDT5-CMNPD20698 and NUDT5-CMNPD24402, a dense cluster was observed. This states that similar conformers of the protein-ligand complexes are present during the simulation, suggesting high stability and low energy (Fig. 5).

However, the scatter plots of the NUDT5-CMNPD20696 and NUDT5-CMNPD19658 complexes were more dispersed and less dense, suggesting that these complexes underwent frequent conformational changes and might be less stable or have high energy. On the other hand, the scatter plot of the reference complex was denser, similar to the first two selected complexes, demonstrating the presence of common conformers with high stability. So, based on the scatter plot analysis, it was observed that the NUDT5-CMNPD20698 and the NUDT5-CMNPD24402 complexes have dense clusters, representing the presence of stable conformers similar to the reference complex, while NUDT5-CMNPD20696 and NUDT5-CMNPD19658 showed the presence of several conformational changes compared to other two and the reference complex.

### Free energy landscape analysis

**Fig. 6:**
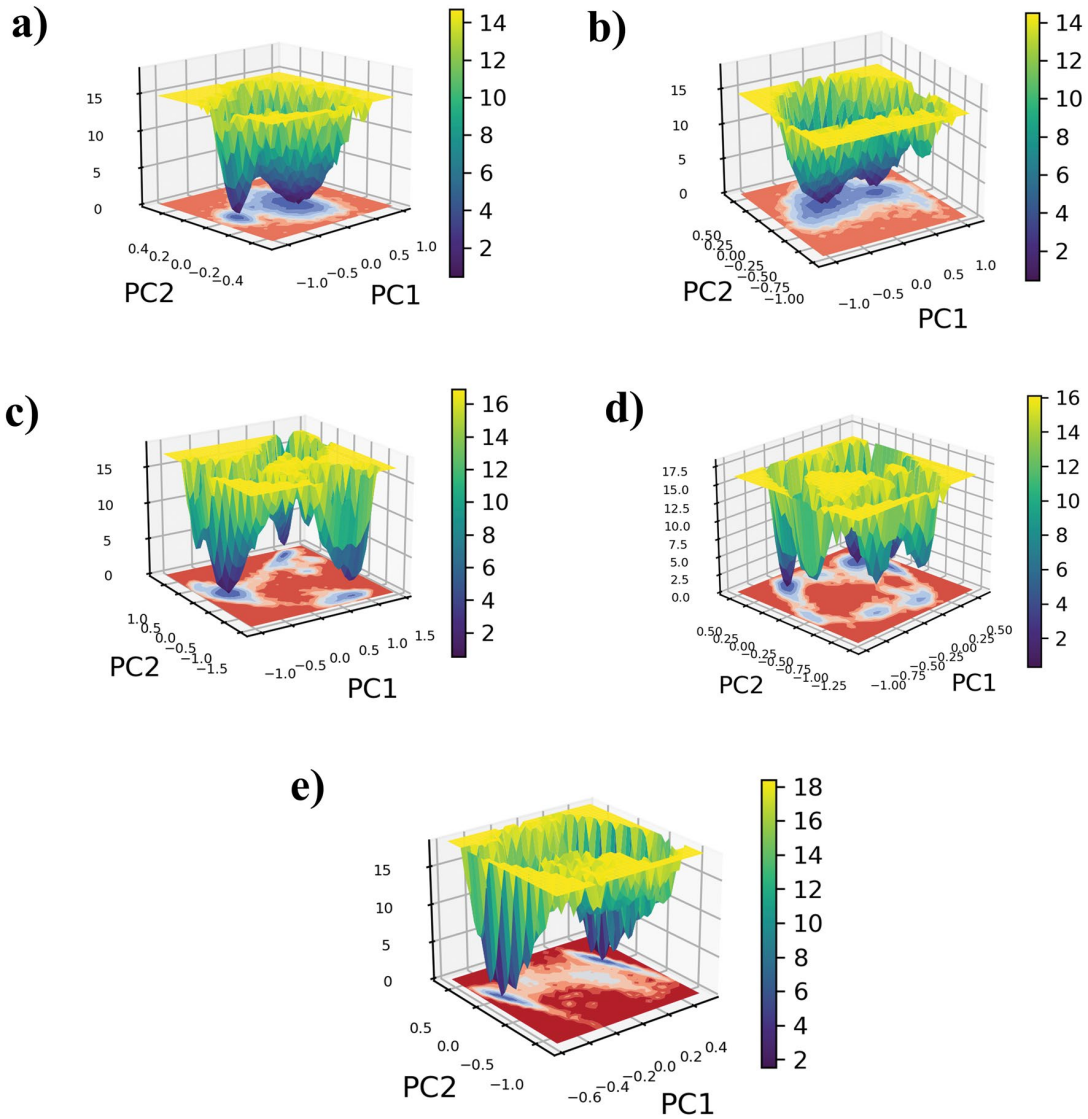
3D FEL plots of the NUDT5 protein docked with the natural compounds **a** CMNPD20698, **b** CMNPD24402, **c** CMNPD20696, **d** CMNPD19658, and the reference compound **e** 958

To gain a deeper understanding of these clusters, we conducted a FEL analysis, which offers an extensive visualization of the clusters. Using PC1 and PC2 to map the clusters onto the energy landscape allows us to explore the energy transition of major motions from each complex obtained by the PCA. The FEL analysis enables the identification of stable conformations and energy transition states, assisting in ligand optimization for enhanced binding affinity. The 3D FEL image provides insights into the presence of conformers structured in narrow or funnel-like formations based on their respective energy levels. Within this 3D representation, the varying color spectrum indicates different ranges of Gibbs energies within each conformer, ranging from minimum to maximum level, illustrating transitions through changes in color intensity. Examining all complexes, including the reference complex, using 3D FEL plots, we observed that minimal energy transitions were represented with dark violet shades, indicating values between 0 and 2 kJ/mol, while maximum energy transitions appeared as bright yellow shades, reaching up to 18 kJ/mol (Fig. 6).

Additionally, basin shading is indicative of stability and reflects energetic dynamics within each complex. The analysis indicated that the selected compounds stabilize the NUDT5 enzyme in a low-energy conformation, supporting their potential as inhibitors. Notably, NUDT5-CMNPD20696 and NUDT5-CMNPD19658, along with the reference complex, displayed wide basins, suggesting frequent energetic fluctuations, whereas NUDT5-CMNPD20698 and NUDT5-CMNPD24402 exhibited narrow basins, suggesting maximum stability with infrequent energetic changes. Based on our comprehensive interpretation of these findings, it was determined that both NUDT5-CMNPD20698 and NUDT5-CMNPD24402 demonstrated superior dynamical stability when compared to the reference complex.

**Fig. 7:**
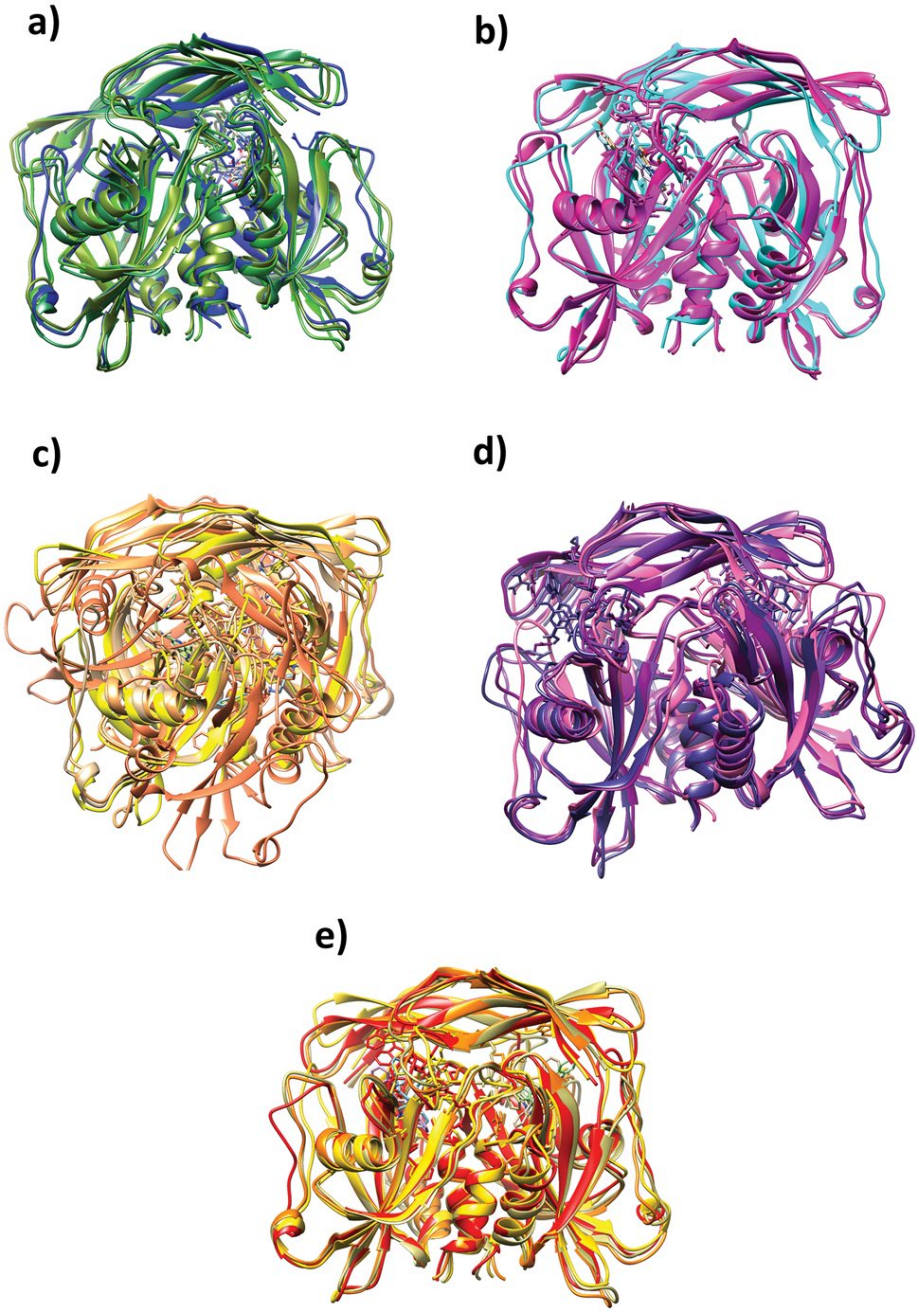
Superimposition of the initial pose with the top-four minimum-energy poses of the **a** NUDT5-CMNPD20698, **b** NUDT5-CMNPD24402, **c** NUDT5-CMNPD20696, **d** NUDT5-CMNPD19658, and **e** the reference NUDT5-958 complexes

To gain a better understanding of the dynamic stability of the complex, four poses from the minimum-energy region were extracted, superimposed, and aligned with the initial poses obtained from the first frame of the simulation trajectory. Based on the obtained RMSD value and structural analysis, the conformational stability of each complex was also analyzed. Superposition of the initial poses with the four minimum-energy poses obtained by FEL showed no conformational changes compared to the initial poses in all complexes. Furthermore, the RMSD value of all the complexes was less than 2.5 Å, suggesting that the complexes are in a stable conformation (Fig. 7 and Figures S1–S5).

### Free binding energy analysis

The Molecular Mechanics/Generalized Born Surface Area (MM/GBSA) method was used to evaluate the strength and stability of the interactions between the protein and the ligands, performing free energy calculations. This approach allows a comparison of binding scores for a selected ligand versus the native ligand when bound to the target protein. In this study, the NUDT5-CMNPD20698, NUDT5-CMNPD24402, NUDT5-CMNPD20696, and NUDT5-CMNPD19658 complexes showed ΔG_total_ energies of − 30.28, − 17.48, − 20.64, and − 22.96 kcal/mol, respectively, whereas the reference complex exhibited a ΔG_total_ energy of − 26.44 kcal/mol. NUDT5-CMNPD20698 showed the highest ΔG_total_ energy among both the selected and reference complexes, indicating stronger binding stability than the others. Further analysis revealed that van der Waals interactions and net gas phase energy play crucial roles in providing maximum stability during the binding process compared to the other components as they contribute significantly more to the total binding free energy, confirming their importance in the affinity of the natural compound with the target protein. Detailed energy components are provided in Table 3.

**Table 3:** MM/GBSA energy components of the docked complexes expressed in kcal/mol

Energy component	NUDT5-CMNPD20698	NUDT5-CMNPD24402	NUDT5-CMNPD20696	NUDT5-CMNPD19658	NUDT5-958
ΔG_total_^a^	− 30.28 ± 2.88	− 17.48 ± 2.85	− 20.64 ± 3.43	− 22.96 ± 4.37	− 26.44 ± 2.46
Van der Waals^b^	− 55.25 ± 2.41	− 42.62 ± 3.42	− 35.55 ± 4.00	− 27.70 ± 5.05	− 42.20 ± 2.18
Electrostatic^c^	− 11.04 ± 5.96	− 9.53 ± 7.58	− 15.23 ± 8.30	− 40.90 ± 15.98	− 32.59 ± 4.63
Polar solvation^d^	42.68 ± 4.94	39.79 ± 4.98	34.64 ± 6.64	50.16 ± 9.73	53.93 ± 3.52
Non-polar solvation^e^	− 6.67 ± 0.24	− 5.13 ± 0.44	− 4.84 ± 0.43	− 4.52 ± 0.41	− 5.59 ± 0.19
Net gas phase^f^	− 44.24 ± 5.70	− 52.15 ± 6.59	− 50.78 ± 8.20	− 68.60 ± 12.69	− 74.79 ± 4.64
Net solvation^g^	21.26 ± 0.44	34.66 ± 5.12	29.97 ± 3.64	45.64 ± 9.89	48.35 ± 3.48

^a^Total binding Gibbs free energy; ^b^contribution of attractive or repulsive forces between molecules or within a single molecule; ^c^contribution of electrostatic interactions between charged particles within molecules; ^d^polar contribution to solvation free energy; ^e^non-polar contribution to solvation free energy; ^f^sum of internal energies of the isolated molecules in vacuum; ^g^energy change when molecules interact with a solvent environment

## Discussion

In this investigation, 2895 natural compounds obtained from marine bacteria were selected from the CMNPD database and subjected to virtual screening against the NUDT5 enzyme, which has been shown to be implicated in cancer aggressiveness, particularly in breast cancer [5, 6, 8]. Through this screening, four potential inhibitors, namely compounds with IDs CMNPD20698 (marinacarboline D), CMNPD24402 (metagenetriindole A), CMNPD20696 (marinacarboline B), and CMNPD19658 (dermacozine C), were identified based on their highly negative docking scores. The re-docking analysis confirmed the strong binding affinity of these compounds with the target protein, suggesting a more detailed investigation to better understand their characteristics. Additionally, the native ligand 958, co-crystallized with the target protein used in this study, was used as a reference compound but exhibited a lower binding score than the selected compounds. Although previously used to identify potential inhibitors for various infections, the use of the CMNPD database in the fight against breast cancer had not yet been explored. Marine natural compounds have great potential as a source for the development of anti-cancer drugs [14, 15]. The identification of marine natural compounds through this in silico investigation could greatly contribute to the future discovery of inhibitors for various other targets. Previously, Tong and colleagues used molecular docking to study the affinity of fourteen organic drugs toward the NUDT5 protein, and their dynamic stability was also assessed through MD simulations and MM/GBSA calculations [38]. Similarly, Almansour utilized computational approaches to identify promising NUDT5 inhibitors [10]. Sultana and colleagues also used molecular docking and virtual screening techniques against the NUDT5 enzyme [39]. These findings confirm that computational methods can play a significant role in future anti-cancer drug discovery to identify novel potential NUDT5 inhibitors.

Molecular interaction analysis of each docked complex showed that Trp^28A^, Trp^46B^, Arg^51A^, and Arg^84A^ participate in key interactions with the ligands. The study conducted by Niranjan and colleagues also found the involvement of these residues in key interactions during the protein surface analysis [40]. The MD simulation trajectory study showed, through RMSD and RMSF analyses, that the binding of the selected natural products stabilizes the protein structure. The stable RMSD values of the selected complexes within the global minimum range (< 0.3 nm) further confirmed the ability of the selected natural compounds to be effective inhibitors against the NUDT5 protein [41]. Furthermore, the RMSF plot analysis of the protein residues in both chains validated that the binding of these natural compounds does not induce any significant conformational changes in the overall protein structure, even after minor residual fluctuations, especially for residues around the 160th position. A similar scenario was observed when the NUDT5 protein was docked with metal complex compounds [11]. The reference compound, 958, also showed a similar pattern during the simulation. By comparing the binding mechanism of the selected ligands to that of the reference compound, it was observed that they all show a similar binding pattern, as confirmed by their dynamic stability.

The PCA and FEL analyses also provided a detailed insight into the dynamic stability of the formed complexes. By analyzing both factors, it was observed the CMNPD20698 and the CMNPD24402 complexes show the highest dynamic stability compared to the other two complexes. In addition, these complexes exhibited somewhat similar dynamic stability to that of the reference complex and displayed minimal energy transition. On the other hand, the CMNPD20696 and the CMNPD19658 complexes showed acceptable stability but the energy transitions were higher than those of the first two and the reference complexes. The PCA plot and FEL plot of the CMNPD20696 and the CMNPD19658 complexes highlighted the presence of high-energy conformers with lower stability. Finally, free binding energy calculations were performed for all the complexes and it was confirmed that the NUDT5-CMNPD20698 complex displayed the highest negative ΔG value, also evidencing a higher affinity compared to the reference complex, with van der Waals interactions being the major contribution. On the other hand, the NUDT5-CMNPD24402 complex showed a higher ΔG value, indicating weaker assembly.

Overall, based on the experimental results, the selected natural compounds displayed strong binding affinity and stability with the NUDT5 protein, similar to the native ligand. The identified natural compounds showed strong potential as NUDT5 inhibitors, with binding scores and interaction patterns similar to known inhibitors. The average RMSD values obtained during the simulations were around 2.5 Å, indicating stable complexes, and comparison with known NUDT5 inhibitors revealed similar stability patterns. The PCA and FEL analyses provided deeper insight into the dynamic stability and conformational behavior of the protein-ligand complexes, confirming their potential inhibitory effect. Additionally, compound CMNPD20698 showed even higher affinity and stability than the native ligand, suggesting its potential ability to strongly inhibit the target protein and deserving its consideration in future in vitro studies.

## Conclusions

The findings of this study highlight the potential of marine natural compounds in drug development and their promising role in targeting the NUDT5 protein. This investigation used computational techniques and energy calculations to study the stability and binding scores of marine natural compounds with drug-like properties with the NUDT5 enzyme, for further therapeutic development. In particular, we utilized an in silico HTVS technique to identify potential NUDT5 inhibitors from natural compounds derived from marine bacteria. Four natural compounds with the best docking scores were selected, and subsequent validation analyses revealed the presence of hydrogen bonds and π–π interactions as driving forces of each complex. The RMSD analysis showed that the complexes have dynamic stability, with an average RMSD value of 0.3 nm, and the RMSF plot demonstrated bond stability with minimal residual fluctuation. The PCA and FEL analyses indicated that the NUDT5-CMNPD20698 complex exhibited the highest conformational stability with minimal transition energy. Additionally, the free binding energy calculations also showed the highest bond stability. However, it is important to note that these findings are based on in silico techniques and further experimental validations are required to confirm the efficacy of the compound CMNPD20698 as an inhibitor of NUDT5. In addition, these compounds should be tested for their pharmacokinetic properties (i.e., absorption, distribution, metabolism, and elimination) as well as for other potential undesired targets. Nevertheless, the results of this study provide valuable information on the potential of natural compounds from marine bacteria as inhibitors of NUDT5, suggesting an in vitro study of these compounds as promising drug candidates or at least as prototypes for the development of novel treatments against NUDT5-related breast cancer.

## Supplementary Information

Below is the link to the electronic supplementary material.

Supplementary file1 (DOCX 4638 KB) Table S1: list of the compounds downloaded from the CMNPD and used for the HTVS study; Figures S1–S5: superimposition of the initial pose of the complex between NUDT5 and the selected natural compounds with the top-four minimum-energy poses and respective structures with minimum global energy

## Data Availability

No datasets were generated or analyzed during the current study.
