# Supplementary material for: Identification of potential NUDT5 inhibitors from marine bacterial natural compounds via molecular dynamics and free energy landscape analysis
Source: Mol Divers. 2024 Sep 3;29(3):1929–44. doi: 10.1007/s11030-024-10950-5 (PMC12081488; doi:10.1007/s11030-024-10950-5)
Supplement: Supplementary file 1 — Supplementary file1 (DOCX 4638 KB) Table S1: list of the compounds downloaded from the CMNPD and used for the HTVS study; Figures S1–S5: superimposition of the initial pose of the complex between NUDT5 and the selected natural compounds with the top-four minimum-energy poses and respective structures with minimum global energy [file 11030_2024_10950_MOESM1_ESM.docx]

**Identification of Potential NUDT5 Inhibitors from Marine Bacterial Natural Compounds via Molecular Dynamics and Free Energy Landscape Analysis**

**Amit Dubey^1,2^, Amer M. Alanazi^3^, Rima Bhardwaj^4🖂^, Andrea Ragusa^5,6🖂^**

1 Department of Pharmacology, Saveetha Dental College, and Hospitals, Saveetha Institute of Medical and Technical Sciences, Saveetha University, Tamil Nādu, Chennai-600077, India; [ameetbioinfo@gmail.com](mailto:ameetbioinfo@gmail.com)

2 Department of Computational Chemistry and Drug Discovery Division, Quanta Calculus, Greater Noida-201310, India

3 Pharmaceutical Biotechnology Laboratory, Department of Pharmaceutical Chemistry, College of Pharmacy, King Saud University, Riyadh, Saudi Arabia; [amalanazi@ksu.edu.sa](mailto:amalanazi@ksu.edu.sa)

4 Department of Chemistry, Poona College, Savitribai Phule Pune University, Pune, India

5 CNR-Nanotec, Institute of Nanotechnology, Via Monteroni, 73100 Lecce, Italy

6 Department of Life Sciences, Health and Health Professions, Link Campus University, Via del Casale di San Pio V 44, 00165 Rome, Italy

***** Correspondence: [bhardwajrima08@gmail.com](mailto:bhardwajrima08@gmail.com) (R.B.); [a.ragusa@unilink.it](mailto:a.ragusa@unilink.it) (A.R.)

Table S1: list of the compounds downloaded from the CMNPD and used for the HTVS study.

| Compound ID | Model ID | Energy (kcal/mol) | nRot |
| --- | --- | --- | --- |
| CMNPD20698 | 1 | -11.2 | 5 |
| CMNPD24402 | 1 | -10.6 | 4 |
| CMNPD20696 | 1 | -10.6 | 6 |
| CMNPD19658 | 1 | -10.5 | 5 |
| CMNPD30148 | 1 | -10.4 | 4 |
| CMNPD20695 | 1 | -10.4 | 6 |
| CMNPD21889 | 1 | -10.1 | 12 |
| CMNPD7990 | 1 | -10.0 | 5 |
| CMNPD17564 | 1 | -9.9 | 11 |
| CMNPD30067 | 1 | -9.9 | 8 |
| CMNPD30153 | 1 | -9.8 | 4 |
| CMNPD15675 | 1 | -9.8 | 4 |
| CMNPD24395 | 1 | -9.8 | 9 |
| CMNPD25856 | 1 | -9.7 | 7 |
| CMNPD23220 | 1 | -9.6 | 7 |
| CMNPD21983 | 1 | -9.5 | 18 |
| CMNPD28614 | 1 | -9.5 | 10 |
| CMNPD23303 | 1 | -9.5 | 6 |
| CMNPD16735 | 1 | -9.5 | 13 |
| CMNPD24426 | 1 | -9.4 | 2 |
| CMNPD8742 | 1 | -9.4 | 13 |
| CMNPD20697 | 1 | -9.4 | 5 |
| CMNPD15676 | 1 | -9.4 | 5 |
| CMNPD23169 | 1 | -9.4 | 4 |
| CMNPD24398 | 1 | -9.4 | 7 |
| CMNPD24394 | 1 | -9.4 | 11 |
| CMNPD23242 | 1 | -9.4 | 3 |
| CMNPD25729 | 1 | -9.3 | 2 |
| CMNPD28643 | 1 | -9.3 | 5 |
| CMNPD17563 | 1 | -9.3 | 9 |
| CMNPD21968 | 1 | -9.3 | 10 |
| CMNPD25855 | 1 | -9.3 | 6 |
| CMNPD23206 | 1 | -9.2 | 9 |
| CMNPD3610 | 1 | -9.2 | 8 |
| CMNPD23227 | 1 | -9.2 | 13 |
| CMNPD24371 | 1 | -9.2 | 9 |
| CMNPD21971 | 1 | -9.2 | 3 |
| CMNPD24448 | 1 | -9.2 | 7 |
| CMNPD27777 | 1 | -9.2 | 7 |
| CMNPD6529 | 1 | -9.1 | 4 |
| CMNPD3611 | 1 | -9.1 | 7 |
| CMNPD27192 | 1 | -9.1 | 8 |
| CMNPD28536 | 1 | -9.1 | 6 |
| CMNPD18668 | 1 | -9.1 | 8 |
| CMNPD28473 | 1 | -9.1 | 9 |
| CMNPD27186 | 1 | -9 | 4 |
| CMNPD20762 | 1 | -9 | 8 |
| CMNPD30086 | 1 | -9 | 10 |
| CMNPD7991 | 1 | -9 | 6 |
| CMNPD6536 | 1 | -9 | 10 |
| CMNPD18649 | 1 | -8.9 | 4 |
| CMNPD13184 | 1 | -8.9 | 5 |
| CMNPD7989 | 1 | -8.9 | 9 |
| CMNPD27119 | 1 | -8.9 | 6 |
| CMNPD19920 | 1 | -8.9 | 17 |
| CMNPD874 | 1 | -8.9 | 4 |
| CMNPD23221 | 1 | -8.9 | 7 |
| CMNPD28487 | 1 | -8.9 | 6 |
| CMNPD24370 | 1 | -8.9 | 8 |
| CMNPD27140 | 1 | -8.9 | 5 |
| CMNPD23161 | 1 | -8.9 | 6 |
| CMNPD19652 | 1 | -8.9 | 3 |
| CMNPD30210 | 1 | -8.9 | 12 |
| CMNPD15679 | 1 | -8.8 | 10 |
| CMNPD28550 | 1 | -8.8 | 4 |
| CMNPD21893 | 1 | -8.8 | 12 |
| CMNPD16546 | 1 | -8.8 | 10 |
| CMNPD24379 | 1 | -8.8 | 8 |
| CMNPD27136 | 1 | -8.8 | 5 |
| CMNPD23311 | 1 | -8.8 | 9 |
| CMNPD24372 | 1 | -8.8 | 9 |
| CMNPD28642 | 1 | -8.8 | 5 |
| CMNPD25731 | 1 | -8.8 | 8 |
| CMNPD17545 | 1 | -8.8 | 3 |
| CMNPD27191 | 1 | -8.8 | 13 |
| CMNPD24428 | 1 | -8.8 | 1 |
| CMNPD19954 | 1 | -8.8 | 6 |
| CMNPD18648 | 1 | -8.8 | 5 |
| CMNPD18634 | 1 | -8.8 | 6 |
| CMNPD14696 | 1 | -8.8 | 10 |
| CMNPD30105 | 1 | -8.8 | 2 |
| CMNPD21900 | 1 | -8.8 | 3 |
| CMNPD27138 | 1 | -8.7 | 4 |
| CMNPD21941 | 1 | -8.7 | 6 |
| CMNPD241 | 1 | -8.7 | 3 |
| CMNPD19722 | 1 | -8.7 | 3 |
| CMNPD12381 | 1 | -8.7 | 13 |
| CMNPD21949 | 1 | -8.7 | 2 |
| CMNPD23168 | 1 | -8.7 | 4 |
| CMNPD6530 | 1 | -8.7 | 2 |
| CMNPD19714 | 1 | -8.7 | 9 |
| CMNPD22308 | 1 | -8.7 | 8 |
| CMNPD19739 | 1 | -8.7 | 3 |
| CMNPD15705 | 1 | -8.7 | 13 |
| CMNPD30150 | 1 | -8.7 | 4 |
| CMNPD24397 | 1 | -8.7 | 10 |
| CMNPD19692 | 1 | -8.7 | 6 |
| CMNPD19716 | 1 | -8.7 | 10 |
| CMNPD30165 | 1 | -8.7 | 8 |
| CMNPD14697 | 1 | -8.7 | 7 |
| CMNPD21982 | 1 | -8.6 | 19 |
| CMNPD5311 | 1 | -8.6 | 8 |
| CMNPD16547 | 1 | -8.6 | 12 |
| CMNPD30104 | 1 | -8.6 | 2 |
| CMNPD21901 | 1 | -8.6 | 5 |
| CMNPD335 | 1 | -8.6 | 2 |
| CMNPD23267 | 1 | -8.6 | 13 |
| CMNPD19738 | 1 | -8.6 | 7 |
| CMNPD28649 | 1 | -8.6 | 8 |
| CMNPD23215 | 1 | -8.6 | 5 |
| CMNPD24449 | 1 | -8.6 | 7 |
| CMNPD30166 | 1 | -8.6 | 8 |
| CMNPD30087 | 1 | -8.6 | 9 |
| CMNPD19735 | 1 | -8.6 | 14 |
| CMNPD28534 | 1 | -8.6 | 8 |
| CMNPD28485 | 1 | -8.6 | 8 |
| CMNPD17544 | 1 | -8.6 | 11 |
| CMNPD19740 | 1 | -8.6 | 3 |
| CMNPD18673 | 1 | -8.6 | 2 |
| CMNPD4645 | 1 | -8.6 | 8 |
| CMNPD27134 | 1 | -8.5 | 7 |
| CMNPD13212 | 1 | -8.5 | 2 |
| CMNPD19674 | 1 | -8.5 | 13 |
| CMNPD25747 | 1 | -8.5 | 9 |
| CMNPD19711 | 1 | -8.5 | 15 |
| CMNPD21891 | 1 | -8.5 | 18 |
| CMNPD18635 | 1 | -8.5 | 3 |
| CMNPD28480 | 1 | -8.5 | 6 |
| CMNPD27797 | 1 | -8.5 | 10 |
| CMNPD21963 | 1 | -8.5 | 8 |
| CMNPD21942 | 1 | -8.5 | 7 |
| CMNPD28488 | 1 | -8.5 | 6 |
| CMNPD13213 | 1 | -8.5 | 4 |
| CMNPD27137 | 1 | -8.4 | 6 |
| CMNPD28479 | 1 | -8.4 | 6 |
| CMNPD19737 | 1 | -8.4 | 8 |
| CMNPD18650 | 1 | -8.4 | 6 |
| CMNPD28507 | 1 | -8.4 | 11 |
| CMNPD19691 | 1 | -8.4 | 6 |
| CMNPD17532 | 1 | -8.4 | 8 |
| CMNPD28481 | 1 | -8.4 | 12 |
| CMNPD17528 | 1 | -8.4 | 7 |
| CMNPD27278 | 1 | -8.4 | 3 |
| CMNPD24392 | 1 | -8.4 | 8 |
| CMNPD25786 | 1 | -8.4 | 1 |
| CMNPD24396 | 1 | -8.4 | 10 |
| CMNPD23231 | 1 | -8.4 | 9 |
| CMNPD25794 | 1 | -8.4 | 4 |
| CMNPD28533 | 1 | -8.4 | 7 |
| CMNPD30149 | 1 | -8.4 | 3 |
| CMNPD28511 | 1 | -8.4 | 5 |
| CMNPD12383 | 1 | -8.4 | 16 |
| CMNPD29956 | 1 | -8.4 | 11 |
| CMNPD25787 | 1 | -8.4 | 1 |
| CMNPD20760 | 1 | -8.4 | 10 |
| CMNPD13214 | 1 | -8.4 | 1 |
| CMNPD20784 | 1 | -8.4 | 3 |
| CMNPD23244 | 1 | -8.4 | 3 |
| CMNPD23149 | 1 | -8.4 | 7 |
| CMNPD25732 | 1 | -8.4 | 11 |
| CMNPD7240 | 1 | -8.4 | 5 |
| CMNPD12380 | 1 | -8.4 | 13 |
| CMNPD10900 | 1 | -8.3 | 6 |
| CMNPD30146 | 1 | -8.3 | 6 |
| CMNPD24353 | 1 | -8.3 | 10 |
| CMNPD19712 | 1 | -8.3 | 8 |
| CMNPD27237 | 1 | -8.3 | 11 |
| CMNPD28458 | 1 | -8.3 | 10 |
| CMNPD21892 | 1 | -8.3 | 13 |
| CMNPD30061 | 1 | -8.3 | 4 |
| CMNPD3798 | 1 | -8.3 | 3 |
| CMNPD30080 | 1 | -8.3 | 13 |
| CMNPD30059 | 1 | -8.3 | 2 |
| CMNPD30214 | 1 | -8.3 | 13 |
| CMNPD18563 | 1 | -8.3 | 4 |
| CMNPD27185 | 1 | -8.3 | 6 |
| CMNPD25882 | 1 | -8.3 | 13 |
| CMNPD19717 | 1 | -8.3 | 14 |
| CMNPD19713 | 1 | -8.3 | 12 |
| CMNPD23610 | 1 | -8.3 | 14 |
| CMNPD28459 | 1 | -8.3 | 9 |
| CMNPD23232 | 1 | -8.3 | 6 |
| CMNPD27232 | 1 | -8.3 | 7 |
| CMNPD30026 | 1 | -8.3 | 4 |
| CMNPD15699 | 1 | -8.3 | 3 |
| CMNPD23204 | 1 | -8.3 | 4 |
| CMNPD21894 | 1 | -8.3 | 15 |
| CMNPD24373 | 1 | -8.3 | 8 |
| CMNPD25785 | 1 | -8.3 | 2 |
| CMNPD24403 | 1 | -8.3 | 1 |
| CMNPD29955 | 1 | -8.3 | 13 |
| CMNPD9393 | 1 | -8.2 | 5 |
| CMNPD15684 | 1 | -8.2 | 8 |
| CMNPD24497 | 1 | -8.2 | 10 |
| CMNPD24956 | 1 | -8.2 | 10 |
| CMNPD30170 | 1 | -8.2 | 13 |
| CMNPD13997 | 1 | -8.2 | 12 |
| CMNPD23240 | 1 | -8.2 | 4 |
| CMNPD24427 | 1 | -8.2 | 2 |
| CMNPD17566 | 1 | -8.2 | 5 |
| CMNPD24362 | 1 | -8.2 | 14 |
| CMNPD23233 | 1 | -8.2 | 8 |
| CMNPD30162 | 1 | -8.2 | 9 |
| CMNPD23229 | 1 | -8.2 | 3 |
| CMNPD25889 | 1 | -8.2 | 15 |
| CMNPD24393 | 1 | -8.2 | 12 |
| CMNPD23266 | 1 | -8.2 | 3 |
| CMNPD27163 | 1 | -8.2 | 4 |
| CMNPD25740 | 1 | -8.2 | 8 |
| CMNPD6535 | 1 | -8.2 | 8 |
| CMNPD24407 | 1 | -8.2 | 1 |
| CMNPD27165 | 1 | -8.2 | 3 |
| CMNPD24494 | 1 | -8.2 | 4 |
| CMNPD21915 | 1 | -8.2 | 8 |
| CMNPD24361 | 1 | -8.2 | 15 |
| CMNPD28510 | 1 | -8.2 | 4 |
| CMNPD6531 | 1 | -8.2 | 2 |
| CMNPD20767 | 1 | -8.2 | 1 |
| CMNPD16544 | 1 | -8.2 | 4 |
| CMNPD28512 | 1 | -8.2 | 5 |
| CMNPD5307 | 1 | -8.2 | 5 |
| CMNPD19653 | 1 | -8.2 | 6 |
| CMNPD28483 | 1 | -8.2 | 5 |
| CMNPD23235 | 1 | -8.2 | 5 |
| CMNPD14699 | 1 | -8.1 | 13 |
| CMNPD21979 | 1 | -8.1 | 17 |
| CMNPD16541 | 1 | -8.1 | 3 |
| CMNPD27215 | 1 | -8.1 | 13 |
| CMNPD27118 | 1 | -8.1 | 2 |
| CMNPD28451 | 1 | -8.1 | 4 |
| CMNPD27275 | 1 | -8.1 | 1 |
| CMNPD15700 | 1 | -8.1 | 3 |
| CMNPD30111 | 1 | -8.1 | 5 |
| CMNPD23241 | 1 | -8.1 | 4 |
| CMNPD19729 | 1 | -8.1 | 4 |
| CMNPD25730 | 1 | -8.1 | 5 |
| CMNPD4648 | 1 | -8.1 | 8 |
| CMNPD22320 | 1 | -8.1 | 8 |
| CMNPD28595 | 1 | -8.1 | 9 |
| CMNPD24496 | 1 | -8.1 | 5 |
| CMNPD15683 | 1 | -8.1 | 9 |
| CMNPD19656 | 1 | -8.1 | 2 |
| CMNPD19715 | 1 | -8.1 | 11 |
| CMNPD23225 | 1 | -8.1 | 2 |
| CMNPD25842 | 1 | -8.1 | 15 |
| CMNPD26399 | 1 | -8.1 | 11 |
| CMNPD24495 | 1 | -8.1 | 4 |
| CMNPD19670 | 1 | -8.1 | 11 |
| CMNPD10904 | 1 | -8.1 | 17 |
| CMNPD27164 | 1 | -8.1 | 4 |
| CMNPD30110 | 1 | -8.1 | 14 |
| CMNPD10871 | 1 | -8.1 | 17 |
| CMNPD10178 | 1 | -8.1 | 12 |
| CMNPD24456 | 1 | -8.1 | 20 |
| CMNPD19709 | 1 | -8.1 | 5 |
| CMNPD325 | 1 | -8.1 | 16 |
| CMNPD25789 | 1 | -8.1 | 4 |
| CMNPD13187 | 1 | -8.1 | 5 |
| CMNPD28460 | 1 | -8.1 | 10 |
| CMNPD24344 | 1 | -8.1 | 10 |
| CMNPD30091 | 1 | -8.1 | 8 |
| CMNPD17560 | 1 | -8.1 | 5 |
| CMNPD4647 | 1 | -8.1 | 8 |
| CMNPD28660 | 1 | -8.1 | 11 |
| CMNPD1749 | 1 | -8.1 | 3 |
| CMNPD16563 | 1 | -8.1 | 12 |
| CMNPD10110 | 1 | -8.1 | 9 |
| CMNPD27174 | 1 | -8.1 | 16 |
| CMNPD28405 | 1 | -8.1 | 2 |
| CMNPD27193 | 1 | -8 | 7 |
| CMNPD30063 | 1 | -8 | 4 |
| CMNPD21043 | 1 | -8 | 13 |
| CMNPD27162 | 1 | -8 | 3 |
| CMNPD20761 | 1 | -8 | 9 |
| CMNPD28606 | 1 | -8 | 13 |
| CMNPD27217 | 1 | -8 | 14 |
| CMNPD30164 | 1 | -8 | 7 |
| CMNPD20673 | 1 | -8 | 4 |
| CMNPD27281 | 1 | -8 | 5 |
| CMNPD23230 | 1 | -8 | 7 |
| CMNPD23243 | 1 | -8 | 2 |
| CMNPD10181 | 1 | -8 | 12 |
| CMNPD30103 | 1 | -8 | 4 |
| CMNPD19668 | 1 | -8 | 8 |
| CMNPD28669 | 1 | -8 | 21 |
| CMNPD10966 | 1 | -8 | 15 |
| CMNPD30107 | 1 | -8 | 14 |
| CMNPD18679 | 1 | -8 | 5 |
| CMNPD28477 | 1 | -8 | 12 |
| CMNPD24957 | 1 | -8 | 11 |
| CMNPD28482 | 1 | -8 | 7 |
| CMNPD9394 | 1 | -8 | 6 |
| CMNPD5308 | 1 | -8 | 8 |
| CMNPD24476 | 1 | -8 | 10 |
| CMNPD30171 | 1 | -7.9 | 2 |
| CMNPD23158 | 1 | -7.9 | 16 |
| CMNPD2561 | 1 | -7.9 | 7 |
| CMNPD24399 | 1 | -7.9 | 4 |
| CMNPD9397 | 1 | -7.9 | 3 |
| CMNPD19710 | 1 | -7.9 | 15 |
| CMNPD12382 | 1 | -7.9 | 16 |
| CMNPD28636 | 1 | -7.9 | 2 |
| CMNPD25887 | 1 | -7.9 | 2 |
| CMNPD28505 | 1 | -7.9 | 7 |
| CMNPD28537 | 1 | -7.9 | 4 |
| CMNPD19726 | 1 | -7.9 | 6 |
| CMNPD17543 | 1 | -7.9 | 4 |
| CMNPD19921 | 1 | -7.9 | 17 |
| CMNPD21948 | 1 | -7.9 | 2 |
| CMNPD25840 | 1 | -7.9 | 17 |
| CMNPD28450 | 1 | -7.9 | 6 |
| CMNPD22305 | 1 | -7.9 | 9 |
| CMNPD21898 | 1 | -7.9 | 6 |
| CMNPD19724 | 1 | -7.9 | 4 |
| CMNPD30084 | 1 | -7.9 | 9 |
| CMNPD27135 | 1 | -7.9 | 6 |
| CMNPD30109 | 1 | -7.9 | 15 |
| CMNPD19687 | 1 | -7.9 | 7 |
| CMNPD23205 | 1 | -7.9 | 3 |
| CMNPD24475 | 1 | -7.9 | 11 |
| CMNPD15678 | 1 | -7.9 | 12 |
| CMNPD27223 | 1 | -7.9 | 3 |
| CMNPD28457 | 1 | -7.9 | 14 |
| CMNPD20672 | 1 | -7.8 | 4 |
| CMNPD28665 | 1 | -7.8 | 9 |
| CMNPD27277 | 1 | -7.8 | 3 |
| CMNPD30064 | 1 | -7.8 | 4 |
| CMNPD15677 | 1 | -7.8 | 12 |
| CMNPD20674 | 1 | -7.8 | 4 |
| CMNPD30112 | 1 | -7.8 | 4 |
| CMNPD27187 | 1 | -7.8 | 3 |
| CMNPD20710 | 1 | -7.8 | 4 |
| CMNPD28516 | 1 | -7.8 | 9 |
| CMNPD18669 | 1 | -7.8 | 3 |
| CMNPD13189 | 1 | -7.8 | 5 |
| CMNPD21946 | 1 | -7.8 | 10 |
| CMNPD21947 | 1 | -7.8 | 11 |
| CMNPD326 | 1 | -7.8 | 16 |
| CMNPD8021 | 1 | -7.8 | 8 |
| CMNPD13190 | 1 | -7.8 | 3 |
| CMNPD27139 | 1 | -7.8 | 3 |
| CMNPD27207 | 1 | -7.8 | 12 |
| CMNPD5310 | 1 | -7.8 | 8 |
| CMNPD5335 | 1 | -7.8 | 5 |
| CMNPD25748 | 1 | -7.8 | 9 |
| CMNPD312 | 1 | -7.8 | 4 |
| CMNPD9390 | 1 | -7.8 | 6 |
| CMNPD27233 | 1 | -7.8 | 13 |
| CMNPD15692 | 1 | -7.8 | 3 |
| CMNPD11663 | 1 | -7.8 | 8 |
| CMNPD14711 | 1 | -7.8 | 10 |
| CMNPD19708 | 1 | -7.8 | 3 |
| CMNPD25817 | 1 | -7.8 | 2 |
| CMNPD28607 | 1 | -7.8 | 7 |
| CMNPD13971 | 1 | -7.8 | 15 |
| CMNPD25829 | 1 | -7.8 | 12 |
| CMNPD7239 | 1 | -7.8 | 5 |
| CMNPD22292 | 1 | -7.8 | 7 |
| CMNPD9459 | 1 | -7.8 | 12 |
| CMNPD27234 | 1 | -7.8 | 12 |
| CMNPD19736 | 1 | -7.8 | 13 |
| CMNPD13254 | 1 | -7.8 | 5 |
| CMNPD24436 | 1 | -7.8 | 6 |
| CMNPD5309 | 1 | -7.8 | 8 |
| CMNPD24864 | 1 | -7.8 | 7 |
| CMNPD27171 | 1 | -7.7 | 3 |
| CMNPD25888 | 1 | -7.7 | 15 |
| CMNPD10108 | 1 | -7.7 | 8 |
| CMNPD15674 | 1 | -7.7 | 2 |
| CMNPD17530 | 1 | -7.7 | 8 |
| CMNPD27229 | 1 | -7.7 | 6 |
| CMNPD8752 | 1 | -7.7 | 4 |
| CMNPD18636 | 1 | -7.7 | 4 |
| CMNPD25845 | 1 | -7.7 | 15 |
| CMNPD27253 | 1 | -7.7 | 4 |
| CMNPD17531 | 1 | -7.7 | 8 |
| CMNPD13976 | 1 | -7.7 | 12 |
| CMNPD30108 | 1 | -7.7 | 15 |
| CMNPD9460 | 1 | -7.7 | 8 |
| CMNPD23208 | 1 | -7.7 | 2 |
| CMNPD25843 | 1 | -7.7 | 19 |
| CMNPD17567 | 1 | -7.7 | 9 |
| CMNPD15696 | 1 | -7.7 | 4 |
| CMNPD13186 | 1 | -7.7 | 13 |
| CMNPD30085 | 1 | -7.7 | 8 |
| CMNPD22306 | 1 | -7.7 | 9 |
| CMNPD27773 | 1 | -7.7 | 9 |
| CMNPD22294 | 1 | -7.7 | 6 |
| CMNPD30060 | 1 | -7.7 | 3 |
| CMNPD25881 | 1 | -7.7 | 13 |
| CMNPD30102 | 1 | -7.7 | 13 |
| CMNPD25841 | 1 | -7.7 | 16 |
| CMNPD13979 | 1 | -7.7 | 11 |
| CMNPD27230 | 1 | -7.7 | 3 |
| CMNPD13973 | 1 | -7.7 | 14 |
| CMNPD27236 | 1 | -7.7 | 12 |
| CMNPD19690 | 1 | -7.7 | 4 |
| CMNPD20680 | 1 | -7.7 | 5 |
| CMNPD19686 | 1 | -7.7 | 6 |
| CMNPD13886 | 1 | -7.7 | 11 |
| CMNPD21943 | 1 | -7.7 | 6 |
| CMNPD20681 | 1 | -7.7 | 5 |
| CMNPD28554 | 1 | -7.7 | 6 |
| CMNPD17604 | 1 | -7.7 | 7 |
| CMNPD28486 | 1 | -7.7 | 11 |
| CMNPD21922 | 1 | -7.7 | 13 |
| CMNPD15682 | 1 | -7.7 | 10 |
| CMNPD23617 | 1 | -7.7 | 9 |
| CMNPD30126 | 1 | -7.7 | 4 |
| CMNPD238 | 1 | -7.6 | 5 |
| CMNPD27235 | 1 | -7.6 | 12 |
| CMNPD25869 | 1 | -7.6 | 3 |
| CMNPD10889 | 1 | -7.6 | 8 |
| CMNPD11664 | 1 | -7.6 | 7 |
| CMNPD19694 | 1 | -7.6 | 10 |
| CMNPD316 | 1 | -7.6 | 4 |
| CMNPD14712 | 1 | -7.6 | 10 |
| CMNPD21964 | 1 | -7.6 | 3 |
| CMNPD21988 | 1 | -7.6 | 5 |
| CMNPD20763 | 1 | -7.6 | 8 |
| CMNPD25819 | 1 | -7.6 | 7 |
| CMNPD24381 | 1 | -7.6 | 6 |
| CMNPD21890 | 1 | -7.6 | 19 |
| CMNPD22293 | 1 | -7.6 | 6 |
| CMNPD27239 | 1 | -7.6 | 25 |
| CMNPD13980 | 1 | -7.6 | 11 |
| CMNPD1024 | 1 | -7.6 | 4 |
| CMNPD11638 | 1 | -7.6 | 6 |
| CMNPD30113 | 1 | -7.6 | 8 |
| CMNPD19723 | 1 | -7.6 | 5 |
| CMNPD28435 | 1 | -7.6 | 11 |
| CMNPD29981 | 1 | -7.6 | 8 |
| CMNPD24498 | 1 | -7.6 | 6 |
| CMNPD15697 | 1 | -7.6 | 4 |
| CMNPD30093 | 1 | -7.6 | 9 |
| CMNPD24525 | 1 | -7.6 | 6 |
| CMNPD24343 | 1 | -7.6 | 10 |
| CMNPD29976 | 1 | -7.6 | 9 |
| CMNPD22291 | 1 | -7.6 | 9 |
| CMNPD20721 | 1 | -7.6 | 3 |
| CMNPD15680 | 1 | -7.6 | 10 |
| CMNPD30088 | 1 | -7.6 | 11 |
| CMNPD29954 | 1 | -7.6 | 16 |
| CMNPD20783 | 1 | -7.6 | 5 |
| CMNPD17586 | 1 | -7.6 | 11 |
| CMNPD17527 | 1 | -7.6 | 7 |
| CMNPD30078 | 1 | -7.6 | 16 |
| CMNPD24977 | 1 | -7.6 | 6 |
| CMNPD9388 | 1 | -7.6 | 9 |
| CMNPD14787 | 1 | -7.6 | 11 |
| CMNPD314 | 1 | -7.6 | 5 |
| CMNPD27231 | 1 | -7.6 | 3 |
| CMNPD14695 | 1 | -7.6 | 8 |
| CMNPD9399 | 1 | -7.6 | 7 |
| CMNPD18677 | 1 | -7.5 | 8 |
| CMNPD15698 | 1 | -7.5 | 5 |
| CMNPD24528 | 1 | -7.5 | 2 |
| CMNPD6532 | 1 | -7.5 | 2 |
| CMNPD17737 | 1 | -7.5 | 14 |
| CMNPD25818 | 1 | -7.5 | 8 |
| CMNPD30169 | 1 | -7.5 | 10 |
| CMNPD25844 | 1 | -7.5 | 14 |
| CMNPD24432 | 1 | -7.5 | 11 |
| CMNPD30045 | 1 | -7.5 | 5 |
| CMNPD19704 | 1 | -7.5 | 4 |
| CMNPD30130 | 1 | -7.5 | 1 |
| CMNPD18645 | 1 | -7.5 | 13 |
| CMNPD8751 | 1 | -7.5 | 5 |
| CMNPD24523 | 1 | -7.5 | 3 |
| CMNPD24342 | 1 | -7.5 | 11 |
| CMNPD17741 | 1 | -7.5 | 18 |
| CMNPD24519 | 1 | -7.5 | 8 |
| CMNPD10115 | 1 | -7.5 | 4 |
| CMNPD29973 | 1 | -7.5 | 7 |
| CMNPD315 | 1 | -7.5 | 2 |
| CMNPD13970 | 1 | -7.5 | 15 |
| CMNPD29951 | 1 | -7.5 | 22 |
| CMNPD21067 | 1 | -7.5 | 8 |
| CMNPD16537 | 1 | -7.5 | 13 |
| CMNPD28599 | 1 | -7.5 | 1 |
| CMNPD23180 | 1 | -7.5 | 6 |
| CMNPD3609 | 1 | -7.5 | 21 |
| CMNPD23281 | 1 | -7.5 | 8 |
| CMNPD27113 | 1 | -7.5 | 3 |
| CMNPD15690 | 1 | -7.5 | 3 |
| CMNPD10109 | 1 | -7.5 | 9 |
| CMNPD14686 | 1 | -7.5 | 14 |
| CMNPD19727 | 1 | -7.5 | 6 |
| CMNPD30211 | 1 | -7.5 | 14 |
| CMNPD16551 | 1 | -7.5 | 14 |
| CMNPD24483 | 1 | -7.5 | 6 |
| CMNPD30168 | 1 | -7.5 | 10 |
| CMNPD239 | 1 | -7.5 | 7 |
| CMNPD22295 | 1 | -7.5 | 7 |
| CMNPD10972 | 1 | -7.5 | 9 |
| CMNPD27117 | 1 | -7.5 | 2 |
| CMNPD28562 | 1 | -7.5 | 5 |
| CMNPD8749 | 1 | -7.5 | 9 |
| CMNPD311 | 1 | -7.5 | 5 |
| CMNPD20734 | 1 | -7.5 | 4 |
| CMNPD10890 | 1 | -7.5 | 8 |
| CMNPD29972 | 1 | -7.5 | 7 |
| CMNPD8788 | 1 | -7.5 | 5 |
| CMNPD9398 | 1 | -7.5 | 4 |
| CMNPD17529 | 1 | -7.5 | 8 |
| CMNPD21976 | 1 | -7.5 | 10 |
| CMNPD18687 | 1 | -7.5 | 10 |
| CMNPD17738 | 1 | -7.5 | 10 |
| CMNPD27256 | 1 | -7.5 | 11 |
| CMNPD25837 | 1 | -7.5 | 15 |
| CMNPD317 | 1 | -7.5 | 5 |
| CMNPD17580 | 1 | -7.5 | 0 |
| CMNPD25846 | 1 | -7.4 | 14 |
| CMNPD14675 | 1 | -7.4 | 4 |
| CMNPD28565 | 1 | -7.4 | 2 |
| CMNPD30209 | 1 | -7.4 | 8 |
| CMNPD27196 | 1 | -7.4 | 10 |
| CMNPD19706 | 1 | -7.4 | 4 |
| CMNPD30097 | 1 | -7.4 | 4 |
| CMNPD5853 | 1 | -7.4 | 7 |
| CMNPD27240 | 1 | -7.4 | 3 |
| CMNPD25820 | 1 | -7.4 | 4 |
| CMNPD24412 | 1 | -7.4 | 7 |
| CMNPD24437 | 1 | -7.4 | 9 |
| CMNPD27279 | 1 | -7.4 | 4 |
| CMNPD24424 | 1 | -7.4 | 3 |
| CMNPD28613 | 1 | -7.4 | 7 |
| CMNPD24478 | 1 | -7.4 | 4 |
| CMNPD15804 | 1 | -7.4 | 10 |
| CMNPD27261 | 1 | -7.4 | 10 |
| CMNPD27257 | 1 | -7.4 | 11 |
| CMNPD10961 | 1 | -7.4 | 11 |
| CMNPD30233 | 1 | -7.4 | 9 |
| CMNPD24518 | 1 | -7.4 | 8 |
| CMNPD23226 | 1 | -7.4 | 6 |
| CMNPD30155 | 1 | -7.4 | 5 |
| CMNPD13188 | 1 | -7.4 | 5 |
| CMNPD16733 | 1 | -7.4 | 3 |
| CMNPD23173 | 1 | -7.4 | 7 |
| CMNPD23253 | 1 | -7.4 | 7 |
| CMNPD28448 | 1 | -7.4 | 18 |
| CMNPD20670 | 1 | -7.4 | 5 |
| CMNPD30129 | 1 | -7.4 | 1 |
| CMNPD28670 | 1 | -7.4 | 10 |
| CMNPD28456 | 1 | -7.4 | 16 |
| CMNPD2570 | 1 | -7.4 | 17 |
| CMNPD27280 | 1 | -7.4 | 10 |
| CMNPD25886 | 1 | -7.4 | 2 |
| CMNPD27258 | 1 | -7.4 | 7 |
| CMNPD24417 | 1 | -7.4 | 2 |
| CMNPD23214 | 1 | -7.4 | 5 |
| CMNPD22304 | 1 | -7.4 | 9 |
| CMNPD20766 | 1 | -7.4 | 1 |
| CMNPD30090 | 1 | -7.4 | 5 |
| CMNPD15691 | 1 | -7.4 | 3 |
| CMNPD1031 | 1 | -7.4 | 3 |
| CMNPD28535 | 1 | -7.4 | 6 |
| CMNPD20671 | 1 | -7.4 | 4 |
| CMNPD1746 | 1 | -7.4 | 8 |
| CMNPD19725 | 1 | -7.4 | 6 |
| CMNPD20765 | 1 | -7.4 | 2 |
| CMNPD18875 | 1 | -7.4 | 14 |
| CMNPD4126 | 1 | -7.4 | 9 |
| CMNPD13978 | 1 | -7.4 | 11 |
| CMNPD28442 | 1 | -7.4 | 13 |
| CMNPD20754 | 1 | -7.3 | 8 |
| CMNPD19669 | 1 | -7.3 | 7 |
| CMNPD17559 | 1 | -7.3 | 2 |
| CMNPD28513 | 1 | -7.3 | 8 |
| CMNPD7993 | 1 | -7.3 | 4 |
| CMNPD5852 | 1 | -7.3 | 7 |
| CMNPD10965 | 1 | -7.3 | 15 |
| CMNPD10887 | 1 | -7.3 | 15 |
| CMNPD28625 | 1 | -7.3 | 21 |
| CMNPD10179 | 1 | -7.3 | 20 |
| CMNPD30154 | 1 | -7.3 | 3 |
| CMNPD23177 | 1 | -7.3 | 8 |
| CMNPD29974 | 1 | -7.3 | 6 |
| CMNPD21028 | 1 | -7.3 | 15 |
| CMNPD18680 | 1 | -7.3 | 5 |
| CMNPD27115 | 1 | -7.3 | 2 |
| CMNPD21974 | 1 | -7.3 | 3 |
| CMNPD19707 | 1 | -7.3 | 3 |
| CMNPD28406 | 1 | -7.3 | 2 |
| CMNPD27205 | 1 | -7.3 | 4 |
| CMNPD5306 | 1 | -7.3 | 5 |
| CMNPD17548 | 1 | -7.3 | 11 |
| CMNPD30160 | 1 | -7.3 | 8 |
| CMNPD30095 | 1 | -7.3 | 4 |
| CMNPD28647 | 1 | -7.3 | 17 |
| CMNPD24455 | 1 | -7.3 | 21 |
| CMNPD28600 | 1 | -7.3 | 1 |
| CMNPD28447 | 1 | -7.3 | 10 |
| CMNPD28440 | 1 | -7.3 | 12 |
| CMNPD19673 | 1 | -7.3 | 3 |
| CMNPD13974 | 1 | -7.3 | 13 |
| CMNPD13972 | 1 | -7.3 | 14 |
| CMNPD19956 | 1 | -7.3 | 10 |
| CMNPD27776 | 1 | -7.3 | 4 |
| CMNPD20669 | 1 | -7.3 | 4 |
| CMNPD23181 | 1 | -7.3 | 8 |
| CMNPD30234 | 1 | -7.3 | 7 |
| CMNPD16536 | 1 | -7.3 | 12 |
| CMNPD23144 | 1 | -7.3 | 4 |
| CMNPD28499 | 1 | -7.3 | 15 |
| CMNPD25788 | 1 | -7.3 | 2 |
| CMNPD23175 | 1 | -7.3 | 9 |
| CMNPD27181 | 1 | -7.3 | 2 |
| CMNPD24491 | 1 | -7.3 | 4 |
| CMNPD28617 | 1 | -7.3 | 5 |
| CMNPD18686 | 1 | -7.3 | 4 |
| CMNPD2192 | 1 | -7.3 | 16 |
| CMNPD24457 | 1 | -7.3 | 7 |
| CMNPD24411 | 1 | -7.3 | 7 |
| CMNPD24490 | 1 | -7.3 | 4 |
| CMNPD23162 | 1 | -7.3 | 6 |
| CMNPD24477 | 1 | -7.3 | 3 |
| CMNPD27238 | 1 | -7.3 | 25 |
| CMNPD29952 | 1 | -7.3 | 11 |
| CMNPD18670 | 1 | -7.3 | 1 |
| CMNPD16564 | 1 | -7.2 | 12 |
| CMNPD29971 | 1 | -7.2 | 6 |
| CMNPD30092 | 1 | -7.2 | 6 |
| CMNPD23160 | 1 | -7.2 | 6 |
| CMNPD28566 | 1 | -7.2 | 3 |
| CMNPD16540 | 1 | -7.2 | 5 |
| CMNPD327 | 1 | -7.2 | 18 |
| CMNPD25868 | 1 | -7.2 | 10 |
| CMNPD12469 | 1 | -7.2 | 10 |
| CMNPD19681 | 1 | -7.2 | 5 |
| CMNPD27172 | 1 | -7.2 | 9 |
| CMNPD19693 | 1 | -7.2 | 10 |
| CMNPD19698 | 1 | -7.2 | 4 |
| CMNPD16573 | 1 | -7.2 | 23 |
| CMNPD20789 | 1 | -7.2 | 4 |
| CMNPD18662 | 1 | -7.2 | 9 |
| CMNPD23219 | 1 | -7.2 | 7 |
| CMNPD20712 | 1 | -7.2 | 3 |
| CMNPD16539 | 1 | -7.2 | 3 |
| CMNPD23145 | 1 | -7.2 | 4 |
| CMNPD30207 | 1 | -7.2 | 12 |
| CMNPD28462 | 1 | -7.2 | 5 |
| CMNPD28572 | 1 | -7.2 | 4 |
| CMNPD30159 | 1 | -7.2 | 6 |
| CMNPD23176 | 1 | -7.2 | 9 |
| CMNPD10886 | 1 | -7.2 | 14 |
| CMNPD23179 | 1 | -7.2 | 7 |
| CMNPD18672 | 1 | -7.2 | 3 |
| CMNPD28506 | 1 | -7.2 | 12 |
| CMNPD28569 | 1 | -7.2 | 6 |
| CMNPD313 | 1 | -7.2 | 5 |
| CMNPD19666 | 1 | -7.2 | 7 |
| CMNPD16538 | 1 | -7.2 | 11 |
| CMNPD26432 | 1 | -7.2 | 1 |
| CMNPD23146 | 1 | -7.2 | 4 |
| CMNPD27248 | 1 | -7.2 | 5 |
| CMNPD20707 | 1 | -7.2 | 4 |
| CMNPD16579 | 1 | -7.2 | 7 |
| CMNPD10962 | 1 | -7.2 | 15 |
| CMNPD18671 | 1 | -7.2 | 1 |
| CMNPD28564 | 1 | -7.2 | 6 |
| CMNPD18683 | 1 | -7.2 | 2 |
| CMNPD11665 | 1 | -7.2 | 10 |
| CMNPD18682 | 1 | -7.2 | 5 |
| CMNPD30161 | 1 | -7.2 | 9 |
| CMNPD19731 | 1 | -7.2 | 4 |
| CMNPD18647 | 1 | -7.2 | 13 |
| CMNPD19672 | 1 | -7.2 | 0 |
| CMNPD13178 | 1 | -7.2 | 5 |
| CMNPD30152 | 1 | -7.2 | 4 |
| CMNPD28608 | 1 | -7.2 | 4 |
| CMNPD19705 | 1 | -7.2 | 4 |
| CMNPD16580 | 1 | -7.2 | 7 |
| CMNPD28455 | 1 | -7.2 | 17 |
| CMNPD25725 | 1 | -7.2 | 2 |
| CMNPD27106 | 1 | -7.2 | 19 |
| CMNPD14786 | 1 | -7.2 | 10 |
| CMNPD30163 | 1 | -7.2 | 5 |
| CMNPD28449 | 1 | -7.2 | 19 |
| CMNPD27114 | 1 | -7.2 | 4 |
| CMNPD17535 | 1 | -7.2 | 4 |
| CMNPD17549 | 1 | -7.2 | 10 |
| CMNPD334 | 1 | -7.2 | 2 |
| CMNPD13207 | 1 | -7.2 | 3 |
| CMNPD20701 | 1 | -7.2 | 7 |
| CMNPD16542 | 1 | -7.2 | 4 |
| CMNPD21989 | 1 | -7.1 | 4 |
| CMNPD17739 | 1 | -7.1 | 18 |
| CMNPD23174 | 1 | -7.1 | 7 |
| CMNPD30158 | 1 | -7.1 | 5 |
| CMNPD24410 | 1 | -7.1 | 6 |
| CMNPD21064 | 1 | -7.1 | 11 |
| CMNPD30098 | 1 | -7.1 | 5 |
| CMNPD23170 | 1 | -7.1 | 7 |
| CMNPD28463 | 1 | -7.1 | 3 |
| CMNPD23255 | 1 | -7.1 | 2 |
| CMNPD30157 | 1 | -7.1 | 6 |
| CMNPD18644 | 1 | -7.1 | 13 |
| CMNPD30099 | 1 | -7.1 | 6 |
| CMNPD28567 | 1 | -7.1 | 4 |
| CMNPD22307 | 1 | -7.1 | 9 |
| CMNPD13192 | 1 | -7.1 | 5 |
| CMNPD18904 | 1 | -7.1 | 13 |
| CMNPD15687 | 1 | -7.1 | 2 |
| CMNPD21913 | 1 | -7.1 | 14 |
| CMNPD30068 | 1 | -7.1 | 4 |
| CMNPD10888 | 1 | -7.1 | 16 |
| CMNPD8019 | 1 | -7.1 | 13 |
| CMNPD2569 | 1 | -7.1 | 16 |
| CMNPD27771 | 1 | -7.1 | 17 |
| CMNPD13892 | 1 | -7.1 | 7 |
| CMNPD8790 | 1 | -7.1 | 15 |
| CMNPD13977 | 1 | -7.1 | 11 |
| CMNPD20723 | 1 | -7.1 | 10 |
| CMNPD25867 | 1 | -7.1 | 10 |
| CMNPD8750 | 1 | -7.1 | 8 |
| CMNPD21945 | 1 | -7.1 | 13 |
| CMNPD24445 | 1 | -7.1 | 7 |
| CMNPD10191 | 1 | -7.1 | 0 |
| CMNPD7236 | 1 | -7.1 | 4 |
| CMNPD25885 | 1 | -7.1 | 15 |
| CMNPD18681 | 1 | -7.1 | 4 |
| CMNPD23217 | 1 | -7.1 | 5 |
| CMNPD14791 | 1 | -7.1 | 23 |
| CMNPD28568 | 1 | -7.1 | 5 |
| CMNPD24338 | 1 | -7.1 | 23 |
| CMNPD19734 | 1 | -7.1 | 9 |
| CMNPD21899 | 1 | -7.1 | 6 |
| CMNPD13975 | 1 | -7.1 | 13 |
| CMNPD28601 | 1 | -7.1 | 2 |
| CMNPD23143 | 1 | -7.1 | 4 |
| CMNPD1745 | 1 | -7.1 | 9 |
| CMNPD13256 | 1 | -7.1 | 4 |
| CMNPD25839 | 1 | -7.1 | 16 |
| CMNPD20682 | 1 | -7.1 | 5 |
| CMNPD29975 | 1 | -7.1 | 8 |
| CMNPD18646 | 1 | -7.1 | 12 |
| CMNPD20705 | 1 | -7.1 | 3 |
| CMNPD30236 | 1 | -7.1 | 16 |
| CMNPD15693 | 1 | -7.1 | 7 |
| CMNPD28461 | 1 | -7.1 | 14 |
| CMNPD240 | 1 | -7.1 | 3 |
| CMNPD24429 | 1 | -7.1 | 13 |
| CMNPD6534 | 1 | -7.1 | 11 |
| CMNPD18678 | 1 | -7.1 | 10 |
| CMNPD24349 | 1 | -7.1 | 3 |
| CMNPD17585 | 1 | -7.1 | 2 |
| CMNPD25727 | 1 | -7.1 | 2 |
| CMNPD23171 | 1 | -7.1 | 8 |
| CMNPD1747 | 1 | -7.1 | 9 |
| CMNPD22322 | 1 | -7.1 | 9 |
| CMNPD17736 | 1 | -7.1 | 12 |
| CMNPD20689 | 1 | -7 | 5 |
| CMNPD25726 | 1 | -7 | 2 |
| CMNPD28592 | 1 | -7 | 7 |
| CMNPD17603 | 1 | -7 | 4 |
| CMNPD22303 | 1 | -7 | 17 |
| CMNPD28441 | 1 | -7 | 12 |
| CMNPD30047 | 1 | -7 | 6 |
| CMNPD24444 | 1 | -7 | 6 |
| CMNPD18685 | 1 | -7 | 4 |
| CMNPD19682 | 1 | -7 | 5 |
| CMNPD27273 | 1 | -7 | 4 |
| CMNPD20706 | 1 | -7 | 2 |
| CMNPD27206 | 1 | -7 | 5 |
| CMNPD19730 | 1 | -7 | 3 |
| CMNPD23188 | 1 | -7 | 5 |
| CMNPD23178 | 1 | -7 | 8 |
| CMNPD16581 | 1 | -7 | 7 |
| CMNPD18684 | 1 | -7 | 3 |
| CMNPD23192 | 1 | -7 | 6 |
| CMNPD28593 | 1 | -7 | 6 |
| CMNPD30215 | 1 | -7 | 6 |
| CMNPD27175 | 1 | -7 | 16 |
| CMNPD24482 | 1 | -7 | 6 |
| CMNPD24359 | 1 | -7 | 6 |
| CMNPD25872 | 1 | -7 | 10 |
| CMNPD27255 | 1 | -7 | 10 |
| CMNPD27251 | 1 | -7 | 4 |
| CMNPD24474 | 1 | -7 | 11 |
| CMNPD9458 | 1 | -7 | 15 |
| CMNPD10971 | 1 | -7 | 2 |
| CMNPD19671 | 1 | -7 | 7 |
| CMNPD10107 | 1 | -7 | 10 |
| CMNPD30212 | 1 | -7 | 15 |
| CMNPD24520 | 1 | -7 | 3 |
| CMNPD328 | 1 | -7 | 10 |
| CMNPD30206 | 1 | -7 | 12 |
| CMNPD20711 | 1 | -7 | 4 |
| CMNPD17546 | 1 | -7 | 11 |
| CMNPD17550 | 1 | -7 | 10 |
| CMNPD17551 | 1 | -7 | 12 |
| CMNPD952 | 1 | -7 | 2 |
| CMNPD7270 | 1 | -7 | 12 |
| CMNPD23172 | 1 | -7 | 8 |
| CMNPD21045 | 1 | -6.9 | 5 |
| CMNPD25873 | 1 | -6.9 | 10 |
| CMNPD16560 | 1 | -6.9 | 4 |
| CMNPD18629 | 1 | -6.9 | 13 |
| CMNPD10901 | 1 | -6.9 | 6 |
| CMNPD24357 | 1 | -6.9 | 7 |
| CMNPD24446 | 1 | -6.9 | 7 |
| CMNPD9396 | 1 | -6.9 | 8 |
| CMNPD16582 | 1 | -6.9 | 5 |
| CMNPD9457 | 1 | -6.9 | 15 |
| CMNPD30096 | 1 | -6.9 | 4 |
| CMNPD23185 | 1 | -6.9 | 4 |
| CMNPD20727 | 1 | -6.9 | 12 |
| CMNPD13202 | 1 | -6.9 | 11 |
| CMNPD28439 | 1 | -6.9 | 15 |
| CMNPD13906 | 1 | -6.9 | 4 |
| CMNPD30128 | 1 | -6.9 | 4 |
| CMNPD25783 | 1 | -6.9 | 3 |
| CMNPD18874 | 1 | -6.9 | 14 |
| CMNPD6537 | 1 | -6.9 | 12 |
| CMNPD28498 | 1 | -6.9 | 9 |
| CMNPD27250 | 1 | -6.9 | 6 |
| CMNPD24493 | 1 | -6.9 | 5 |
| CMNPD25883 | 1 | -6.9 | 14 |
| CMNPD27252 | 1 | -6.9 | 8 |
| CMNPD23184 | 1 | -6.9 | 4 |
| CMNPD23183 | 1 | -6.9 | 3 |
| CMNPD30046 | 1 | -6.9 | 4 |
| CMNPD23157 | 1 | -6.9 | 17 |
| CMNPD27259 | 1 | -6.9 | 10 |
| CMNPD27220 | 1 | -6.9 | 2 |
| CMNPD28594 | 1 | -6.9 | 7 |
| CMNPD321 | 1 | -6.9 | 7 |
| CMNPD28541 | 1 | -6.9 | 5 |
| CMNPD28612 | 1 | -6.9 | 7 |
| CMNPD19657 | 1 | -6.9 | 4 |
| CMNPD28476 | 1 | -6.9 | 13 |
| CMNPD28630 | 1 | -6.9 | 17 |
| CMNPD22319 | 1 | -6.9 | 12 |
| CMNPD30083 | 1 | -6.9 | 4 |
| CMNPD20764 | 1 | -6.9 | 3 |
| CMNPD7992 | 1 | -6.9 | 7 |
| CMNPD21070 | 1 | -6.9 | 15 |
| CMNPD24484 | 1 | -6.9 | 4 |
| CMNPD13267 | 1 | -6.9 | 15 |
| CMNPD30118 | 1 | -6.9 | 9 |
| CMNPD28464 | 1 | -6.9 | 3 |
| CMNPD23616 | 1 | -6.9 | 14 |
| CMNPD27249 | 1 | -6.9 | 6 |
| CMNPD16543 | 1 | -6.9 | 6 |
| CMNPD28514 | 1 | -6.9 | 2 |
| CMNPD18630 | 1 | -6.9 | 14 |
| CMNPD24435 | 1 | -6.9 | 12 |
| CMNPD10190 | 1 | -6.9 | 0 |
| CMNPD24479 | 1 | -6.9 | 15 |
| CMNPD10189 | 1 | -6.9 | 6 |
| CMNPD25874 | 1 | -6.9 | 8 |
| CMNPD20702 | 1 | -6.9 | 7 |
| CMNPD27260 | 1 | -6.9 | 9 |
| CMNPD19667 | 1 | -6.9 | 6 |
| CMNPD25749 | 1 | -6.9 | 9 |
| CMNPD7271 | 1 | -6.9 | 12 |
| CMNPD29959 | 1 | -6.9 | 16 |
| CMNPD24521 | 1 | -6.9 | 2 |
| CMNPD24976 | 1 | -6.9 | 7 |
| CMNPD23210 | 1 | -6.9 | 2 |
| CMNPD30131 | 1 | -6.9 | 2 |
| CMNPD27772 | 1 | -6.9 | 17 |
| CMNPD24360 | 1 | -6.9 | 7 |
| CMNPD20745 | 1 | -6.9 | 14 |
| CMNPD28443 | 1 | -6.9 | 4 |
| CMNPD7232 | 1 | -6.8 | 4 |
| CMNPD20713 | 1 | -6.8 | 5 |
| CMNPD25822 | 1 | -6.8 | 5 |
| CMNPD25756 | 1 | -6.8 | 17 |
| CMNPD14788 | 1 | -6.8 | 10 |
| CMNPD7278 | 1 | -6.8 | 2 |
| CMNPD21916 | 1 | -6.8 | 10 |
| CMNPD10892 | 1 | -6.8 | 8 |
| CMNPD17552 | 1 | -6.8 | 12 |
| CMNPD24960 | 1 | -6.8 | 19 |
| CMNPD25838 | 1 | -6.8 | 17 |
| CMNPD25759 | 1 | -6.8 | 15 |
| CMNPD25761 | 1 | -6.8 | 16 |
| CMNPD9395 | 1 | -6.8 | 8 |
| CMNPD23238 | 1 | -6.8 | 6 |
| CMNPD4646 | 1 | -6.8 | 9 |
| CMNPD18631 | 1 | -6.8 | 15 |
| CMNPD16545 | 1 | -6.8 | 5 |
| CMNPD16578 | 1 | -6.8 | 10 |
| CMNPD27274 | 1 | -6.8 | 2 |
| CMNPD16577 | 1 | -6.8 | 10 |
| CMNPD10174 | 1 | -6.8 | 12 |
| CMNPD23186 | 1 | -6.8 | 2 |
| CMNPD27267 | 1 | -6.8 | 3 |
| CMNPD310 | 1 | -6.8 | 8 |
| CMNPD13208 | 1 | -6.8 | 2 |
| CMNPD23189 | 1 | -6.8 | 5 |
| CMNPD6560 | 1 | -6.8 | 12 |
| CMNPD23159 | 1 | -6.8 | 7 |
| CMNPD16571 | 1 | -6.8 | 13 |
| CMNPD29980 | 1 | -6.8 | 5 |
| CMNPD2571 | 1 | -6.8 | 8 |
| CMNPD2193 | 1 | -6.8 | 17 |
| CMNPD13904 | 1 | -6.8 | 5 |
| CMNPD29963 | 1 | -6.8 | 18 |
| CMNPD14687 | 1 | -6.8 | 2 |
| CMNPD27269 | 1 | -6.8 | 4 |
| CMNPD23209 | 1 | -6.8 | 3 |
| CMNPD10116 | 1 | -6.7 | 12 |
| CMNPD30156 | 1 | -6.7 | 7 |
| CMNPD4125 | 1 | -6.7 | 10 |
| CMNPD27272 | 1 | -6.7 | 4 |
| CMNPD13198 | 1 | -6.7 | 11 |
| CMNPD25744 | 1 | -6.7 | 10 |
| CMNPD13200 | 1 | -6.7 | 11 |
| CMNPD9402 | 1 | -6.7 | 21 |
| CMNPD23236 | 1 | -6.7 | 10 |
| CMNPD13203 | 1 | -6.7 | 11 |
| CMNPD28521 | 1 | -6.7 | 1 |
| CMNPD28563 | 1 | -6.7 | 4 |
| CMNPD24431 | 1 | -6.7 | 12 |
| CMNPD11645 | 1 | -6.7 | 4 |
| CMNPD25871 | 1 | -6.7 | 1 |
| CMNPD27130 | 1 | -6.7 | 7 |
| CMNPD14789 | 1 | -6.7 | 27 |
| CMNPD20782 | 1 | -6.7 | 4 |
| CMNPD27116 | 1 | -6.7 | 3 |
| CMNPD7233 | 1 | -6.7 | 3 |
| CMNPD30203 | 1 | -6.7 | 12 |
| CMNPD13210 | 1 | -6.7 | 2 |
| CMNPD30119 | 1 | -6.7 | 25 |
| CMNPD19732 | 1 | -6.7 | 5 |
| CMNPD13266 | 1 | -6.7 | 15 |
| CMNPD9400 | 1 | -6.7 | 3 |
| CMNPD24434 | 1 | -6.7 | 12 |
| CMNPD28508 | 1 | -6.7 | 14 |
| CMNPD25760 | 1 | -6.7 | 17 |
| CMNPD10192 | 1 | -6.7 | 0 |
| CMNPD24350 | 1 | -6.7 | 2 |
| CMNPD7272 | 1 | -6.7 | 16 |
| CMNPD29977 | 1 | -6.7 | 8 |
| CMNPD17553 | 1 | -6.7 | 12 |
| CMNPD27219 | 1 | -6.7 | 1 |
| CMNPD13982 | 1 | -6.7 | 23 |
| CMNPD10893 | 1 | -6.7 | 8 |
| CMNPD13903 | 1 | -6.7 | 3 |
| CMNPD24451 | 1 | -6.7 | 9 |
| CMNPD27271 | 1 | -6.7 | 4 |
| CMNPD25746 | 1 | -6.7 | 4 |
| CMNPD28628 | 1 | -6.7 | 19 |
| CMNPD11722 | 1 | -6.7 | 14 |
| CMNPD30039 | 1 | -6.7 | 5 |
| CMNPD16572 | 1 | -6.7 | 13 |
| CMNPD23182 | 1 | -6.7 | 3 |
| CMNPD20686 | 1 | -6.7 | 3 |
| CMNPD13891 | 1 | -6.7 | 1 |
| CMNPD24447 | 1 | -6.7 | 8 |
| CMNPD23187 | 1 | -6.7 | 4 |
| CMNPD4668 | 1 | -6.7 | 15 |
| CMNPD30235 | 1 | -6.7 | 19 |
| CMNPD25764 | 1 | -6.6 | 15 |
| CMNPD30122 | 1 | -6.6 | 5 |
| CMNPD25884 | 1 | -6.6 | 14 |
| CMNPD28579 | 1 | -6.6 | 12 |
| CMNPD17556 | 1 | -6.6 | 8 |
| CMNPD10105 | 1 | -6.6 | 27 |
| CMNPD10891 | 1 | -6.6 | 8 |
| CMNPD19926 | 1 | -6.6 | 16 |
| CMNPD29970 | 1 | -6.6 | 20 |
| CMNPD875 | 1 | -6.6 | 1 |
| CMNPD25755 | 1 | -6.6 | 18 |
| CMNPD26396 | 1 | -6.6 | 20 |
| CMNPD237 | 1 | -6.6 | 2 |
| CMNPD14700 | 1 | -6.6 | 5 |
| CMNPD28644 | 1 | -6.6 | 13 |
| CMNPD13894 | 1 | -6.6 | 4 |
| CMNPD19733 | 1 | -6.6 | 4 |
| CMNPD25743 | 1 | -6.6 | 9 |
| CMNPD28538 | 1 | -6.6 | 4 |
| CMNPD28626 | 1 | -6.6 | 21 |
| CMNPD23237 | 1 | -6.6 | 6 |
| CMNPD17547 | 1 | -6.6 | 12 |
| CMNPD27133 | 1 | -6.6 | 3 |
| CMNPD17554 | 1 | -6.6 | 7 |
| CMNPD30044 | 1 | -6.6 | 4 |
| CMNPD19662 | 1 | -6.6 | 3 |
| CMNPD21987 | 1 | -6.6 | 4 |
| CMNPD20725 | 1 | -6.6 | 17 |
| CMNPD28637 | 1 | -6.6 | 1 |
| CMNPD30079 | 1 | -6.6 | 16 |
| CMNPD16732 | 1 | -6.6 | 15 |
| CMNPD25762 | 1 | -6.6 | 15 |
| CMNPD28434 | 1 | -6.6 | 9 |
| CMNPD16554 | 1 | -6.6 | 14 |
| CMNPD2195 | 1 | -6.6 | 15 |
| CMNPD30232 | 1 | -6.6 | 8 |
| CMNPD27132 | 1 | -6.6 | 3 |
| CMNPD24358 | 1 | -6.6 | 7 |
| CMNPD8789 | 1 | -6.6 | 1 |
| CMNPD27173 | 1 | -6.6 | 6 |
| CMNPD20687 | 1 | -6.6 | 2 |
| CMNPD24347 | 1 | -6.6 | 3 |
| CMNPD13268 | 1 | -6.6 | 15 |
| CMNPD25821 | 1 | -6.6 | 9 |
| CMNPD28620 | 1 | -6.6 | 7 |
| CMNPD28509 | 1 | -6.6 | 5 |
| CMNPD17537 | 1 | -6.6 | 2 |
| CMNPD11643 | 1 | -6.6 | 8 |
| CMNPD13255 | 1 | -6.6 | 17 |
| CMNPD21930 | 1 | -6.6 | 7 |
| CMNPD21929 | 1 | -6.6 | 7 |
| CMNPD10131 | 1 | -6.5 | 17 |
| CMNPD28478 | 1 | -6.5 | 13 |
| CMNPD30127 | 1 | -6.5 | 3 |
| CMNPD28426 | 1 | -6.5 | 4 |
| CMNPD21069 | 1 | -6.5 | 16 |
| CMNPD7273 | 1 | -6.5 | 16 |
| CMNPD22263 | 1 | -6.5 | 8 |
| CMNPD17721 | 1 | -6.5 | 18 |
| CMNPD30142 | 1 | -6.5 | 1 |
| CMNPD13209 | 1 | -6.5 | 1 |
| CMNPD27199 | 1 | -6.5 | 4 |
| CMNPD25765 | 1 | -6.5 | 14 |
| CMNPD10894 | 1 | -6.5 | 7 |
| CMNPD25758 | 1 | -6.5 | 16 |
| CMNPD953 | 1 | -6.5 | 1 |
| CMNPD29962 | 1 | -6.5 | 18 |
| CMNPD10104 | 1 | -6.5 | 28 |
| CMNPD5331 | 1 | -6.5 | 13 |
| CMNPD15799 | 1 | -6.5 | 7 |
| CMNPD5304 | 1 | -6.5 | 20 |
| CMNPD17720 | 1 | -6.5 | 19 |
| CMNPD28556 | 1 | -6.5 | 1 |
| CMNPD20688 | 1 | -6.5 | 11 |
| CMNPD30025 | 1 | -6.5 | 4 |
| CMNPD17740 | 1 | -6.5 | 18 |
| CMNPD28695 | 1 | -6.5 | 15 |
| CMNPD20768 | 1 | -6.5 | 2 |
| CMNPD23274 | 1 | -6.5 | 12 |
| CMNPD8787 | 1 | -6.5 | 16 |
| CMNPD28427 | 1 | -6.5 | 5 |
| CMNPD27276 | 1 | -6.5 | 6 |
| CMNPD25757 | 1 | -6.5 | 16 |
| CMNPD7234 | 1 | -6.5 | 3 |
| CMNPD28639 | 1 | -6.5 | 2 |
| CMNPD30089 | 1 | -6.5 | 5 |
| CMNPD7235 | 1 | -6.5 | 3 |
| CMNPD3608 | 1 | -6.5 | 21 |
| CMNPD28651 | 1 | -6.5 | 19 |
| CMNPD14790 | 1 | -6.5 | 25 |
| CMNPD24418 | 1 | -6.5 | 3 |
| CMNPD30213 | 1 | -6.5 | 16 |
| CMNPD29953 | 1 | -6.5 | 3 |
| CMNPD27243 | 1 | -6.5 | 1 |
| CMNPD24489 | 1 | -6.5 | 4 |
| CMNPD11642 | 1 | -6.5 | 7 |
| CMNPD11723 | 1 | -6.5 | 14 |
| CMNPD14676 | 1 | -6.5 | 3 |
| CMNPD25815 | 1 | -6.4 | 6 |
| CMNPD18688 | 1 | -6.4 | 9 |
| CMNPD872 | 1 | -6.4 | 11 |
| CMNPD8737 | 1 | -6.4 | 16 |
| CMNPD28686 | 1 | -6.4 | 3 |
| CMNPD13968 | 1 | -6.4 | 15 |
| CMNPD10903 | 1 | -6.4 | 16 |
| CMNPD30114 | 1 | -6.4 | 7 |
| CMNPD27266 | 1 | -6.4 | 3 |
| CMNPD27105 | 1 | -6.4 | 19 |
| CMNPD30062 | 1 | -6.4 | 3 |
| CMNPD30048 | 1 | -6.4 | 4 |
| CMNPD24459 | 1 | -6.4 | 11 |
| CMNPD2194 | 1 | -6.4 | 16 |
| CMNPD14701 | 1 | -6.4 | 6 |
| CMNPD27791 | 1 | -6.4 | 3 |
| CMNPD25763 | 1 | -6.4 | 16 |
| CMNPD27268 | 1 | -6.4 | 3 |
| CMNPD17539 | 1 | -6.4 | 6 |
| CMNPD28539 | 1 | -6.4 | 3 |
| CMNPD7237 | 1 | -6.4 | 3 |
| CMNPD24421 | 1 | -6.4 | 18 |
| CMNPD11639 | 1 | -6.4 | 10 |
| CMNPD28549 | 1 | -6.4 | 5 |
| CMNPD15694 | 1 | -6.4 | 6 |
| CMNPD24460 | 1 | -6.4 | 11 |
| CMNPD11641 | 1 | -6.4 | 7 |
| CMNPD7995 | 1 | -6.4 | 3 |
| CMNPD29978 | 1 | -6.4 | 9 |
| CMNPD324 | 1 | -6.4 | 14 |
| CMNPD27264 | 1 | -6.4 | 3 |
| CMNPD4654 | 1 | -6.4 | 1 |
| CMNPD23276 | 1 | -6.4 | 12 |
| CMNPD16558 | 1 | -6.4 | 3 |
| CMNPD27263 | 1 | -6.4 | 2 |
| CMNPD25742 | 1 | -6.4 | 2 |
| CMNPD23268 | 1 | -6.4 | 16 |
| CMNPD7277 | 1 | -6.4 | 5 |
| CMNPD8753 | 1 | -6.4 | 2 |
| CMNPD23275 | 1 | -6.4 | 12 |
| CMNPD25728 | 1 | -6.4 | 0 |
| CMNPD9468 | 1 | -6.4 | 17 |
| CMNPD7274 | 1 | -6.4 | 17 |
| CMNPD28437 | 1 | -6.3 | 20 |
| CMNPD24865 | 1 | -6.3 | 7 |
| CMNPD14702 | 1 | -6.3 | 6 |
| CMNPD28525 | 1 | -6.3 | 10 |
| CMNPD25860 | 1 | -6.3 | 1 |
| CMNPD13199 | 1 | -6.3 | 11 |
| CMNPD8754 | 1 | -6.3 | 8 |
| CMNPD13895 | 1 | -6.3 | 5 |
| CMNPD28524 | 1 | -6.3 | 10 |
| CMNPD11657 | 1 | -6.3 | 11 |
| CMNPD29979 | 1 | -6.3 | 10 |
| CMNPD26411 | 1 | -6.3 | 16 |
| CMNPD7279 | 1 | -6.3 | 6 |
| CMNPD14704 | 1 | -6.3 | 6 |
| CMNPD14689 | 1 | -6.3 | 14 |
| CMNPD30082 | 1 | -6.3 | 3 |
| CMNPD28682 | 1 | -6.3 | 16 |
| CMNPD21912 | 1 | -6.3 | 15 |
| CMNPD21902 | 1 | -6.3 | 3 |
| CMNPD19927 | 1 | -6.3 | 17 |
| CMNPD7266 | 1 | -6.3 | 6 |
| CMNPD13197 | 1 | -6.3 | 11 |
| CMNPD320 | 1 | -6.3 | 13 |
| CMNPD28526 | 1 | -6.3 | 12 |
| CMNPD25816 | 1 | -6.3 | 7 |
| CMNPD13273 | 1 | -6.3 | 19 |
| CMNPD8741 | 1 | -6.3 | 7 |
| CMNPD21986 | 1 | -6.3 | 3 |
| CMNPD27197 | 1 | -6.3 | 3 |
| CMNPD28631 | 1 | -6.3 | 19 |
| CMNPD3601 | 1 | -6.3 | 0 |
| CMNPD27198 | 1 | -6.3 | 3 |
| CMNPD27200 | 1 | -6.3 | 3 |
| CMNPD28519 | 1 | -6.3 | 1 |
| CMNPD23234 | 1 | -6.3 | 4 |
| CMNPD22285 | 1 | -6.3 | 20 |
| CMNPD30032 | 1 | -6.3 | 4 |
| CMNPD30081 | 1 | -6.3 | 4 |
| CMNPD24433 | 1 | -6.3 | 10 |
| CMNPD23216 | 1 | -6.3 | 5 |
| CMNPD329 | 1 | -6.2 | 12 |
| CMNPD28438 | 1 | -6.2 | 19 |
| CMNPD28527 | 1 | -6.2 | 7 |
| CMNPD28605 | 1 | -6.2 | 11 |
| CMNPD27265 | 1 | -6.2 | 4 |
| CMNPD876 | 1 | -6.2 | 1 |
| CMNPD28597 | 1 | -6.2 | 1 |
| CMNPD23273 | 1 | -6.2 | 12 |
| CMNPD13278 | 1 | -6.2 | 18 |
| CMNPD13279 | 1 | -6.2 | 19 |
| CMNPD11720 | 1 | -6.2 | 13 |
| CMNPD28528 | 1 | -6.2 | 6 |
| CMNPD2197 | 1 | -6.2 | 8 |
| CMNPD11714 | 1 | -6.2 | 10 |
| CMNPD27270 | 1 | -6.2 | 3 |
| CMNPD15695 | 1 | -6.2 | 1 |
| CMNPD20691 | 1 | -6.2 | 8 |
| CMNPD20690 | 1 | -6.2 | 11 |
| CMNPD9456 | 1 | -6.2 | 12 |
| CMNPD13907 | 1 | -6.2 | 1 |
| CMNPD17557 | 1 | -6.2 | 13 |
| CMNPD22278 | 1 | -6.2 | 4 |
| CMNPD4131 | 1 | -6.2 | 15 |
| CMNPD14705 | 1 | -6.2 | 7 |
| CMNPD18659 | 1 | -6.2 | 9 |
| CMNPD21040 | 1 | -6.2 | 20 |
| CMNPD28681 | 1 | -6.2 | 19 |
| CMNPD24416 | 1 | -6.2 | 1 |
| CMNPD11644 | 1 | -6.2 | 4 |
| CMNPD28523 | 1 | -6.2 | 8 |
| CMNPD19651 | 1 | -6.2 | 4 |
| CMNPD24425 | 1 | -6.2 | 3 |
| CMNPD24422 | 1 | -6.2 | 15 |
| CMNPD26417 | 1 | -6.2 | 2 |
| CMNPD18562 | 1 | -6.2 | 5 |
| CMNPD29967 | 1 | -6.2 | 17 |
| CMNPD11724 | 1 | -6.2 | 10 |
| CMNPD318 | 1 | -6.1 | 3 |
| CMNPD24346 | 1 | -6.1 | 3 |
| CMNPD15689 | 1 | -6.1 | 3 |
| CMNPD27222 | 1 | -6.1 | 4 |
| CMNPD24369 | 1 | -6.1 | 0 |
| CMNPD28674 | 1 | -6.1 | 13 |
| CMNPD11640 | 1 | -6.1 | 17 |
| CMNPD25724 | 1 | -6.1 | 2 |
| CMNPD954 | 1 | -6.1 | 0 |
| CMNPD29960 | 1 | -6.1 | 17 |
| CMNPD22302 | 1 | -6.1 | 17 |
| CMNPD28454 | 1 | -6.1 | 8 |
| CMNPD27244 | 1 | -6.1 | 1 |
| CMNPD24481 | 1 | -6.1 | 8 |
| CMNPD28432 | 1 | -6.1 | 9 |
| CMNPD24348 | 1 | -6.1 | 3 |
| CMNPD4650 | 1 | -6.1 | 4 |
| CMNPD24450 | 1 | -6.1 | 3 |
| CMNPD27121 | 1 | -6.1 | 8 |
| CMNPD24461 | 1 | -6.1 | 11 |
| CMNPD13275 | 1 | -6.1 | 16 |
| CMNPD13276 | 1 | -6.1 | 21 |
| CMNPD17536 | 1 | -6.1 | 4 |
| CMNPD7238 | 1 | -6.1 | 2 |
| CMNPD27107 | 1 | -6.1 | 19 |
| CMNPD21026 | 1 | -6.1 | 18 |
| CMNPD18870 | 1 | -6.1 | 18 |
| CMNPD29969 | 1 | -6.1 | 16 |
| CMNPD10969 | 1 | -6.1 | 19 |
| CMNPD15688 | 1 | -6.1 | 2 |
| CMNPD14703 | 1 | -6.1 | 7 |
| CMNPD30204 | 1 | -6.1 | 11 |
| CMNPD7269 | 1 | -6.1 | 3 |
| CMNPD25828 | 1 | -6 | 4 |
| CMNPD21041 | 1 | -6 | 21 |
| CMNPD19718 | 1 | -6 | 3 |
| CMNPD16559 | 1 | -6 | 4 |
| CMNPD7276 | 1 | -6 | 6 |
| CMNPD26398 | 1 | -6 | 19 |
| CMNPD19659 | 1 | -6 | 4 |
| CMNPD24430 | 1 | -6 | 13 |
| CMNPD28522 | 1 | -6 | 8 |
| CMNPD28650 | 1 | -6 | 18 |
| CMNPD25827 | 1 | -6 | 14 |
| CMNPD25831 | 1 | -6 | 2 |
| CMNPD28666 | 1 | -6 | 21 |
| CMNPD29964 | 1 | -6 | 15 |
| CMNPD20709 | 1 | -6 | 4 |
| CMNPD27108 | 1 | -6 | 20 |
| CMNPD30071 | 1 | -6 | 1 |
| CMNPD19928 | 1 | -6 | 3 |
| CMNPD13277 | 1 | -6 | 19 |
| CMNPD28484 | 1 | -6 | 2 |
| CMNPD29965 | 1 | -6 | 18 |
| CMNPD19661 | 1 | -6 | 2 |
| CMNPD28696 | 1 | -6 | 18 |
| CMNPD24522 | 1 | -6 | 3 |
| CMNPD22280 | 1 | -6 | 9 |
| CMNPD10182 | 1 | -6 | 20 |
| CMNPD16568 | 1 | -6 | 1 |
| CMNPD19719 | 1 | -6 | 4 |
| CMNPD11646 | 1 | -6 | 4 |
| CMNPD13274 | 1 | -6 | 20 |
| CMNPD11725 | 1 | -6 | 11 |
| CMNPD23203 | 1 | -6 | 7 |
| CMNPD23280 | 1 | -5.9 | 3 |
| CMNPD28436 | 1 | -5.9 | 20 |
| CMNPD19680 | 1 | -5.9 | 5 |
| CMNPD8740 | 1 | -5.9 | 31 |
| CMNPD28652 | 1 | -5.9 | 20 |
| CMNPD13204 | 1 | -5.9 | 11 |
| CMNPD23263 | 1 | -5.9 | 2 |
| CMNPD24420 | 1 | -5.9 | 18 |
| CMNPD30002 | 1 | -5.9 | 6 |
| CMNPD28424 | 1 | -5.9 | 4 |
| CMNPD28429 | 1 | -5.9 | 6 |
| CMNPD10171 | 1 | -5.9 | 11 |
| CMNPD13201 | 1 | -5.9 | 11 |
| CMNPD16556 | 1 | -5.9 | 0 |
| CMNPD28520 | 1 | -5.9 | 4 |
| CMNPD21914 | 1 | -5.9 | 15 |
| CMNPD26397 | 1 | -5.9 | 20 |
| CMNPD7268 | 1 | -5.9 | 1 |
| CMNPD28684 | 1 | -5.9 | 4 |
| CMNPD28570 | 1 | -5.9 | 5 |
| CMNPD27184 | 1 | -5.9 | 20 |
| CMNPD873 | 1 | -5.9 | 11 |
| CMNPD8748 | 1 | -5.9 | 10 |
| CMNPD27168 | 1 | -5.8 | 8 |
| CMNPD27177 | 1 | -5.8 | 9 |
| CMNPD19918 | 1 | -5.8 | 8 |
| CMNPD29968 | 1 | -5.8 | 17 |
| CMNPD25795 | 1 | -5.8 | 4 |
| CMNPD28685 | 1 | -5.8 | 3 |
| CMNPD21911 | 1 | -5.8 | 14 |
| CMNPD29958 | 1 | -5.8 | 20 |
| CMNPD29966 | 1 | -5.8 | 17 |
| CMNPD5303 | 1 | -5.8 | 21 |
| CMNPD21042 | 1 | -5.8 | 10 |
| CMNPD28687 | 1 | -5.8 | 3 |
| CMNPD28683 | 1 | -5.8 | 3 |
| CMNPD18608 | 1 | -5.8 | 18 |
| CMNPD23615 | 1 | -5.8 | 9 |
| CMNPD28555 | 1 | -5.8 | 4 |
| CMNPD342 | 1 | -5.8 | 2 |
| CMNPD2564 | 1 | -5.8 | 6 |
| CMNPD21975 | 1 | -5.8 | 13 |
| CMNPD30065 | 1 | -5.8 | 10 |
| CMNPD22279 | 1 | -5.8 | 10 |
| CMNPD21944 | 1 | -5.8 | 5 |
| CMNPD16553 | 1 | -5.7 | 15 |
| CMNPD24423 | 1 | -5.7 | 16 |
| CMNPD10902 | 1 | -5.7 | 1 |
| CMNPD11648 | 1 | -5.7 | 9 |
| CMNPD30116 | 1 | -5.7 | 8 |
| CMNPD27131 | 1 | -5.7 | 6 |
| CMNPD23609 | 1 | -5.7 | 18 |
| CMNPD24355 | 1 | -5.7 | 5 |
| CMNPD30117 | 1 | -5.7 | 10 |
| CMNPD15685 | 1 | -5.7 | 4 |
| CMNPD28425 | 1 | -5.7 | 4 |
| CMNPD28667 | 1 | -5.7 | 21 |
| CMNPD30030 | 1 | -5.7 | 3 |
| CMNPD30066 | 1 | -5.7 | 10 |
| CMNPD25862 | 1 | -5.7 | 2 |
| CMNPD25861 | 1 | -5.6 | 2 |
| CMNPD17568 | 1 | -5.6 | 8 |
| CMNPD24419 | 1 | -5.6 | 16 |
| CMNPD26412 | 1 | -5.6 | 17 |
| CMNPD10172 | 1 | -5.6 | 24 |
| CMNPD8738 | 1 | -5.6 | 30 |
| CMNPD4653 | 1 | -5.6 | 1 |
| CMNPD30237 | 1 | -5.6 | 2 |
| CMNPD30115 | 1 | -5.6 | 10 |
| CMNPD30106 | 1 | -5.6 | 2 |
| CMNPD29961 | 1 | -5.6 | 18 |
| CMNPD15673 | 1 | -5.6 | 8 |
| CMNPD20724 | 1 | -5.6 | 11 |
| CMNPD24378 | 1 | -5.6 | 0 |
| CMNPD19699 | 1 | -5.6 | 6 |
| CMNPD21044 | 1 | -5.5 | 3 |
| CMNPD24400 | 1 | -5.5 | 4 |
| CMNPD28618 | 1 | -5.5 | 2 |
| CMNPD4652 | 1 | -5.5 | 0 |
| CMNPD28428 | 1 | -5.5 | 4 |
| CMNPD30205 | 1 | -5.5 | 19 |
| CMNPD20677 | 1 | -5.5 | 6 |
| CMNPD7282 | 1 | -5.5 | 3 |
| CMNPD29982 | 1 | -5.5 | 30 |
| CMNPD16557 | 1 | -5.5 | 0 |
| CMNPD11713 | 1 | -5.5 | 10 |
| CMNPD23207 | 1 | -5.5 | 10 |
| CMNPD18663 | 1 | -5.5 | 11 |
| CMNPD21990 | 1 | -5.5 | 1 |
| CMNPD15672 | 1 | -5.5 | 7 |
| CMNPD21977 | 1 | -5.5 | 10 |
| CMNPD28571 | 1 | -5.5 | 5 |
| CMNPD28430 | 1 | -5.5 | 3 |
| CMNPD28560 | 1 | -5.5 | 5 |
| CMNPD16574 | 1 | -5.5 | 29 |
| CMNPD10970 | 1 | -5.5 | 19 |
| CMNPD20699 | 1 | -5.5 | 6 |
| CMNPD7267 | 1 | -5.5 | 4 |
| CMNPD27178 | 1 | -5.5 | 6 |
| CMNPD9401 | 1 | -5.5 | 15 |
| CMNPD4130 | 1 | -5.5 | 1 |
| CMNPD28578 | 1 | -5.5 | 4 |
| CMNPD18623 | 1 | -5.5 | 8 |
| CMNPD25750 | 1 | -5.4 | 9 |
| CMNPD11721 | 1 | -5.4 | 13 |
| CMNPD28529 | 1 | -5.4 | 7 |
| CMNPD20726 | 1 | -5.4 | 13 |
| CMNPD28446 | 1 | -5.4 | 11 |
| CMNPD27254 | 1 | -5.4 | 5 |
| CMNPD30094 | 1 | -5.4 | 0 |
| CMNPD28596 | 1 | -5.4 | 0 |
| CMNPD30218 | 1 | -5.4 | 14 |
| CMNPD28530 | 1 | -5.4 | 8 |
| CMNPD10173 | 1 | -5.4 | 23 |
| CMNPD20722 | 1 | -5.4 | 16 |
| CMNPD28452 | 1 | -5.4 | 3 |
| CMNPD10899 | 1 | -5.3 | 5 |
| CMNPD10180 | 1 | -5.3 | 19 |
| CMNPD30217 | 1 | -5.3 | 8 |
| CMNPD24389 | 1 | -5.3 | 16 |
| CMNPD24328 | 1 | -5.3 | 3 |
| CMNPD28407 | 1 | -5.3 | 2 |
| CMNPD24380 | 1 | -5.3 | 1 |
| CMNPD19677 | 1 | -5.3 | 5 |
| CMNPD28474 | 1 | -5.3 | 10 |
| CMNPD4651 | 1 | -5.3 | 1 |
| CMNPD10114 | 1 | -5.3 | 4 |
| CMNPD16552 | 1 | -5.3 | 15 |
| CMNPD30134 | 1 | -5.3 | 4 |
| CMNPD11712 | 1 | -5.3 | 10 |
| CMNPD319 | 1 | -5.3 | 19 |
| CMNPD26406 | 1 | -5.2 | 10 |
| CMNPD25832 | 1 | -5.2 | 29 |
| CMNPD24367 | 1 | -5.2 | 5 |
| CMNPD15670 | 1 | -5.2 | 6 |
| CMNPD25792 | 1 | -5.2 | 9 |
| CMNPD6528 | 1 | -5.2 | 2 |
| CMNPD29957 | 1 | -5.2 | 19 |
| CMNPD28453 | 1 | -5.2 | 3 |
| CMNPD30038 | 1 | -5.2 | 9 |
| CMNPD23212 | 1 | -5.2 | 9 |
| CMNPD24366 | 1 | -5.2 | 5 |
| CMNPD28408 | 1 | -5.2 | 2 |
| CMNPD11726 | 1 | -5.2 | 11 |
| CMNPD28680 | 1 | -5.2 | 20 |
| CMNPD24415 | 1 | -5.2 | 2 |
| CMNPD30031 | 1 | -5.1 | 4 |
| CMNPD18658 | 1 | -5.1 | 3 |
| CMNPD26404 | 1 | -5.1 | 15 |
| CMNPD13969 | 1 | -5.1 | 16 |
| CMNPD25823 | 1 | -5.1 | 9 |
| CMNPD25824 | 1 | -5.1 | 9 |
| CMNPD21883 | 1 | -5.1 | 29 |
| CMNPD19728 | 1 | -5.1 | 8 |
| CMNPD10905 | 1 | -5.1 | 1 |
| CMNPD24368 | 1 | -5.1 | 5 |
| CMNPD28551 | 1 | -5.1 | 10 |
| CMNPD18665 | 1 | -5.1 | 13 |
| CMNPD18667 | 1 | -5.1 | 10 |
| CMNPD28470 | 1 | -5.1 | 2 |
| CMNPD27176 | 1 | -5 | 9 |
| CMNPD25825 | 1 | -5 | 9 |
| CMNPD28471 | 1 | -5 | 17 |
| CMNPD30219 | 1 | -5 | 16 |
| CMNPD24414 | 1 | -5 | 2 |
| CMNPD26403 | 1 | -5 | 16 |
| CMNPD18624 | 1 | -5 | 5 |
| CMNPD17583 | 1 | -5 | 18 |
| CMNPD10129 | 1 | -5 | 3 |
| CMNPD10967 | 1 | -5 | 21 |
| CMNPD20755 | 1 | -5 | 17 |
| CMNPD25790 | 1 | -4.9 | 4 |
| CMNPD9392 | 1 | -4.9 | 3 |
| CMNPD23302 | 1 | -4.9 | 9 |
| CMNPD14713 | 1 | -4.9 | 1 |
| CMNPD25793 | 1 | -4.9 | 9 |
| CMNPD18664 | 1 | -4.9 | 12 |
| CMNPD28632 | 1 | -4.8 | 20 |
| CMNPD13967 | 1 | -4.8 | 16 |
| CMNPD21896 | 1 | -4.8 | 8 |
| CMNPD18666 | 1 | -4.8 | 13 |
| CMNPD8739 | 1 | -4.8 | 29 |
| CMNPD7986 | 1 | -4.8 | 16 |
| CMNPD5329 | 1 | -4.8 | WARNING: |
| CMNPD6562 | 1 | -4.8 | WARNING: |
| CMNPD25866 | 1 | -4.8 | 12 |
| CMNPD26391 | 1 | -4.8 | 2 |
| CMNPD2196 | 1 | -4.8 | 17 |
| CMNPD27111 | 1 | -4.8 | 11 |
| CMNPD2568 | 1 | -4.8 | 1 |
| CMNPD7281 | 1 | -4.8 | 1 |
| CMNPD13300 | 1 | -4.8 | 5 |
| CMNPD15671 | 1 | -4.8 | 6 |
| CMNPD21904 | 1 | -4.8 | 9 |
| CMNPD6539 | 1 | -4.8 | 21 |
| CMNPD20675 | 1 | -4.8 | 6 |
| CMNPD24970 | 1 | -4.8 | 9 |
| CMNPD24971 | 1 | -4.8 | 8 |
| CMNPD16585 | 1 | -4.7 | 0 |
| CMNPD23265 | 1 | -4.7 | 8 |
| CMNPD29983 | 1 | -4.7 | 30 |
| CMNPD21027 | 1 | -4.7 | 19 |
| CMNPD28423 | 1 | -4.7 | 4 |
| CMNPD18657 | 1 | -4.7 | 3 |
| CMNPD19649 | 1 | -4.7 | 7 |
| CMNPD7231 | 1 | -4.7 | 20 |
| CMNPD27183 | 1 | -4.7 | 23 |
| CMNPD20676 | 1 | -4.7 | 6 |
| CMNPD24969 | 1 | -4.7 | 9 |
| CMNPD17526 | 1 | -4.7 | 19 |
| CMNPD30221 | 1 | -4.7 | 15 |
| CMNPD20678 | 1 | -4.6 | 6 |
| CMNPD19647 | 1 | -4.6 | 7 |
| CMNPD30123 | 1 | -4.6 | 3 |
| CMNPD23254 | 1 | -4.6 | 7 |
| CMNPD10102 | 1 | -4.6 | 32 |
| CMNPD10898 | 1 | -4.6 | 5 |
| CMNPD9391 | 1 | -4.6 | 4 |
| CMNPD28629 | 1 | -4.6 | 20 |
| CMNPD16586 | 1 | -4.6 | 0 |
| CMNPD23211 | 1 | -4.6 | 9 |
| CMNPD323 | 1 | -4.6 | 21 |
| CMNPD21910 | 1 | -4.6 | 14 |
| CMNPD4655 | 1 | -4.5 | 2 |
| CMNPD1748 | 1 | -4.5 | 3 |
| CMNPD30052 | 1 | -4.5 | 8 |
| CMNPD7229 | 1 | -4.5 | 32 |
| CMNPD5328 | 1 | -4.5 | WARNING: |
| CMNPD19660 | 1 | -4.5 | 2 |
| CMNPD23163 | 1 | -4.5 | 1 |
| CMNPD4127 | 1 | -4.5 | 1 |
| CMNPD21903 | 1 | -4.4 | 4 |
| CMNPD17540 | 1 | -4.4 | 7 |
| CMNPD4669 | 1 | -4.4 | 1 |
| CMNPD25833 | 1 | -4.4 | 31 |
| CMNPD28515 | 1 | -4.4 | 1 |
| CMNPD10874 | 1 | -4.4 | 31 |
| CMNPD20708 | 1 | -4.4 | 5 |
| CMNPD8745 | 1 | -4.4 | 21 |
| CMNPD30208 | 1 | -4.4 | 15 |
| CMNPD23213 | 1 | -4.4 | 9 |
| CMNPD25939 | 1 | -4.4 | 4 |
| CMNPD28422 | 1 | -4.4 | 3 |
| CMNPD23228 | 1 | -4.3 | 7 |
| CMNPD27120 | 1 | -4.3 | 8 |
| CMNPD25806 | 1 | -4.3 | 8 |
| CMNPD24413 | 1 | -4.3 | 0 |
| CMNPD341 | 1 | -4.3 | 2 |
| CMNPD19695 | 1 | -4.3 | 8 |
| CMNPD5856 | 1 | -4.3 | 21 |
| CMNPD28635 | 1 | -4.2 | 20 |
| CMNPD30220 | 1 | -4.2 | 16 |
| CMNPD18661 | 1 | -4.2 | 11 |
| CMNPD16587 | 1 | -4.2 | 0 |
| CMNPD18652 | 1 | -4.2 | 6 |
| CMNPD16590 | 1 | -4.2 | 0 |
| CMNPD15801 | 1 | -4.1 | 7 |
| CMNPD20757 | 1 | -4.1 | 6 |
| CMNPD18654 | 1 | -4.1 | 6 |
| CMNPD21961 | 1 | -4 | 2 |
| CMNPD18903 | 1 | -4 | 21 |


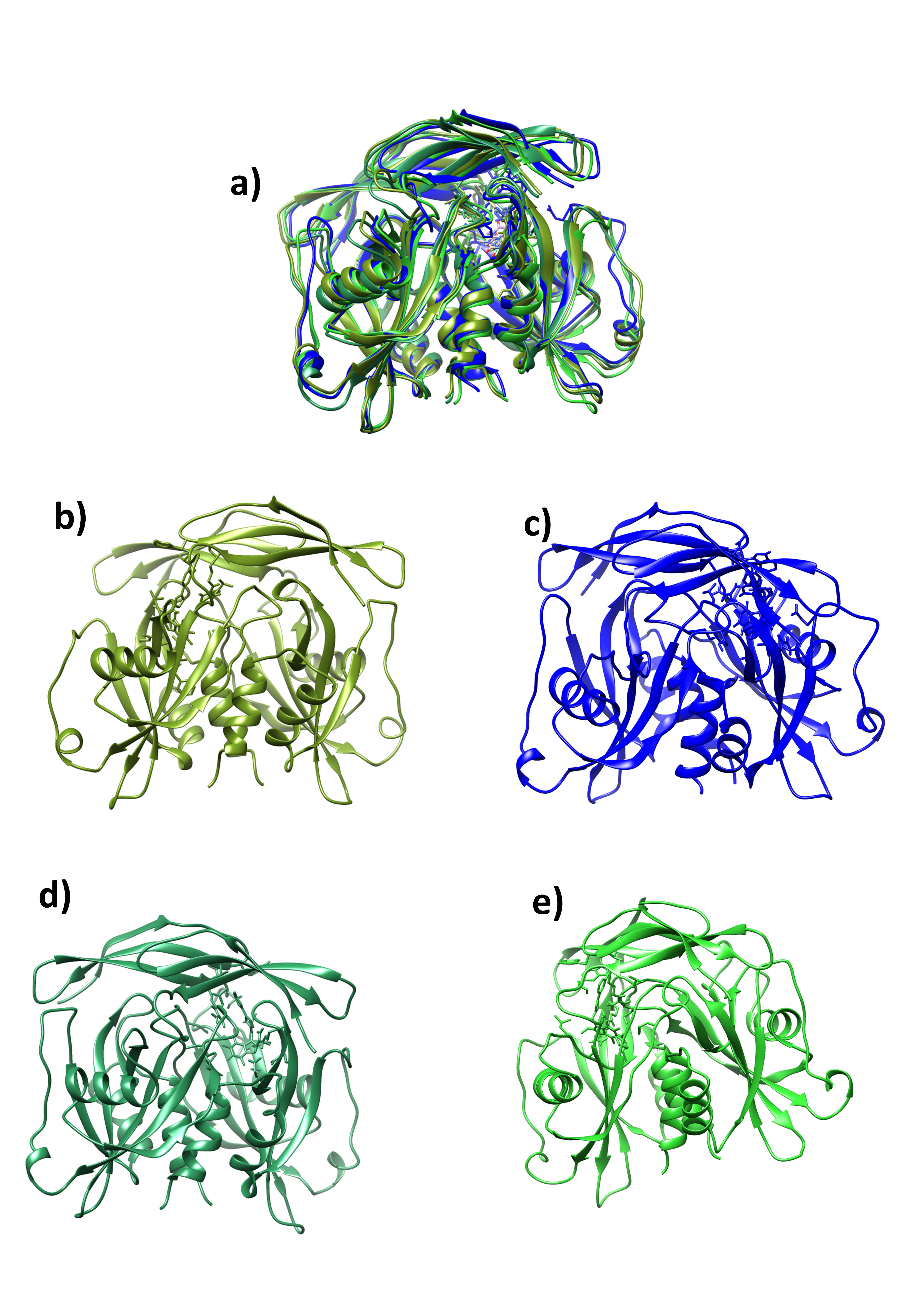


**Figure S1.** (a) Superimposition of the initial pose of the NUDT5-CMNPD20698 complex with the top-four minimum-energy poses. (b, c, d, and e) Respective structures with minimum global energy.


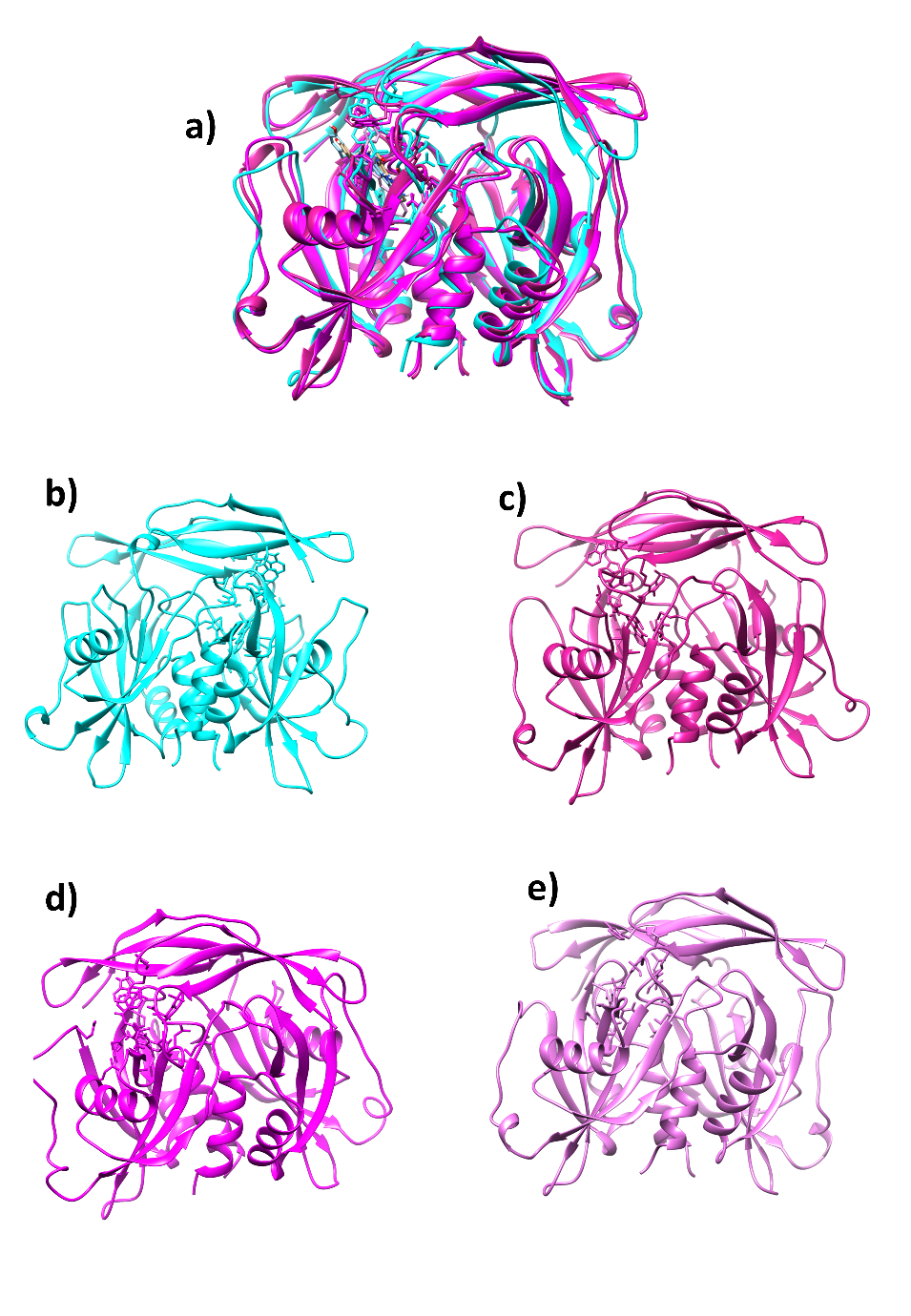


**Figure S2.** (a) Superimposition of the initial pose of the NUDT5-CMNPD24402 complex with the top-four minimum-energy poses. (b, c, d, and e) Respective structures with minimum global energy.


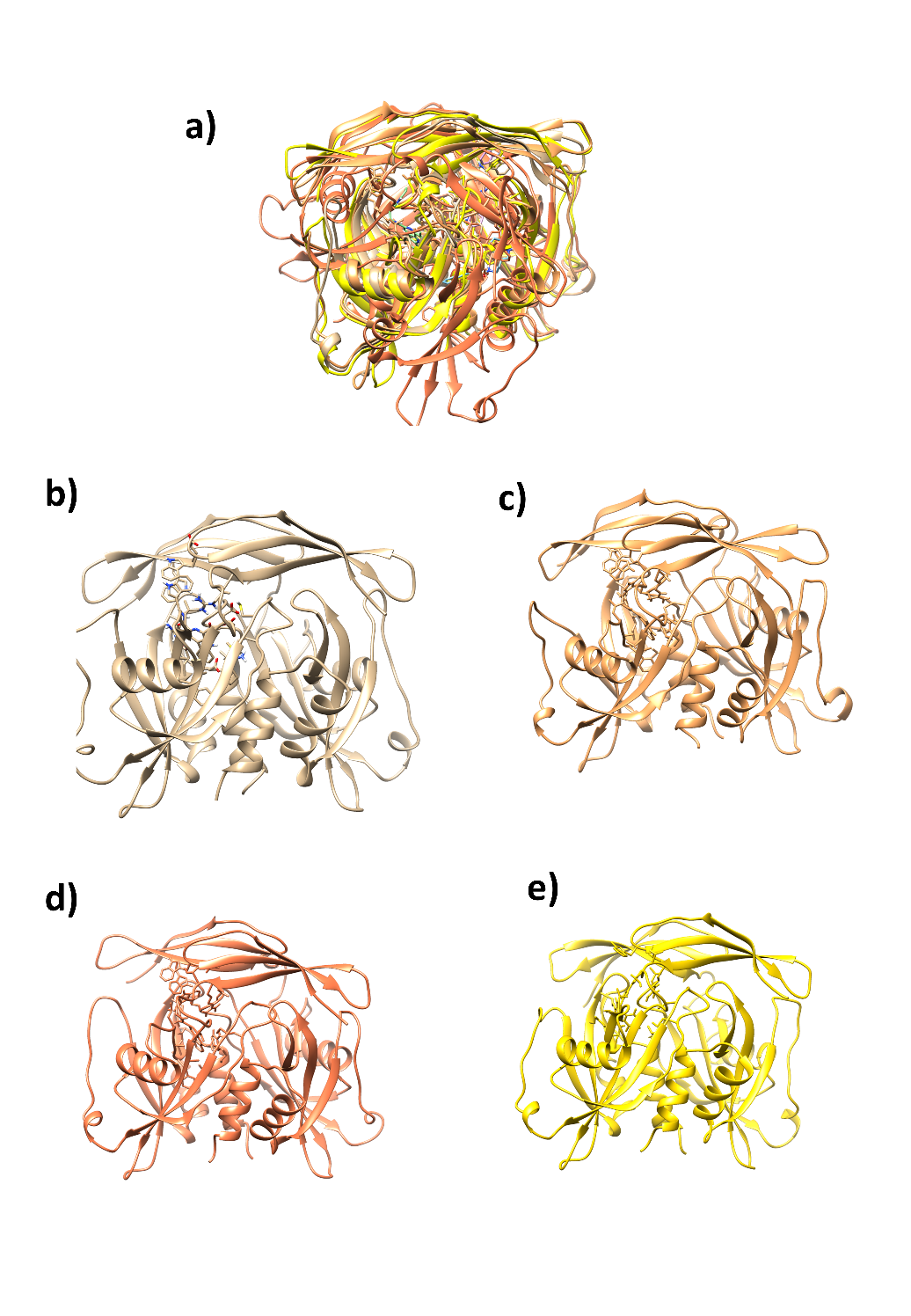


**Figure S3.** (a) Superimposition of the initial pose of the NUDT5-CMNPD20696 complex with the top-four minimum-energy poses. (b, c, d, and e) Respective structures with minimum global energy.


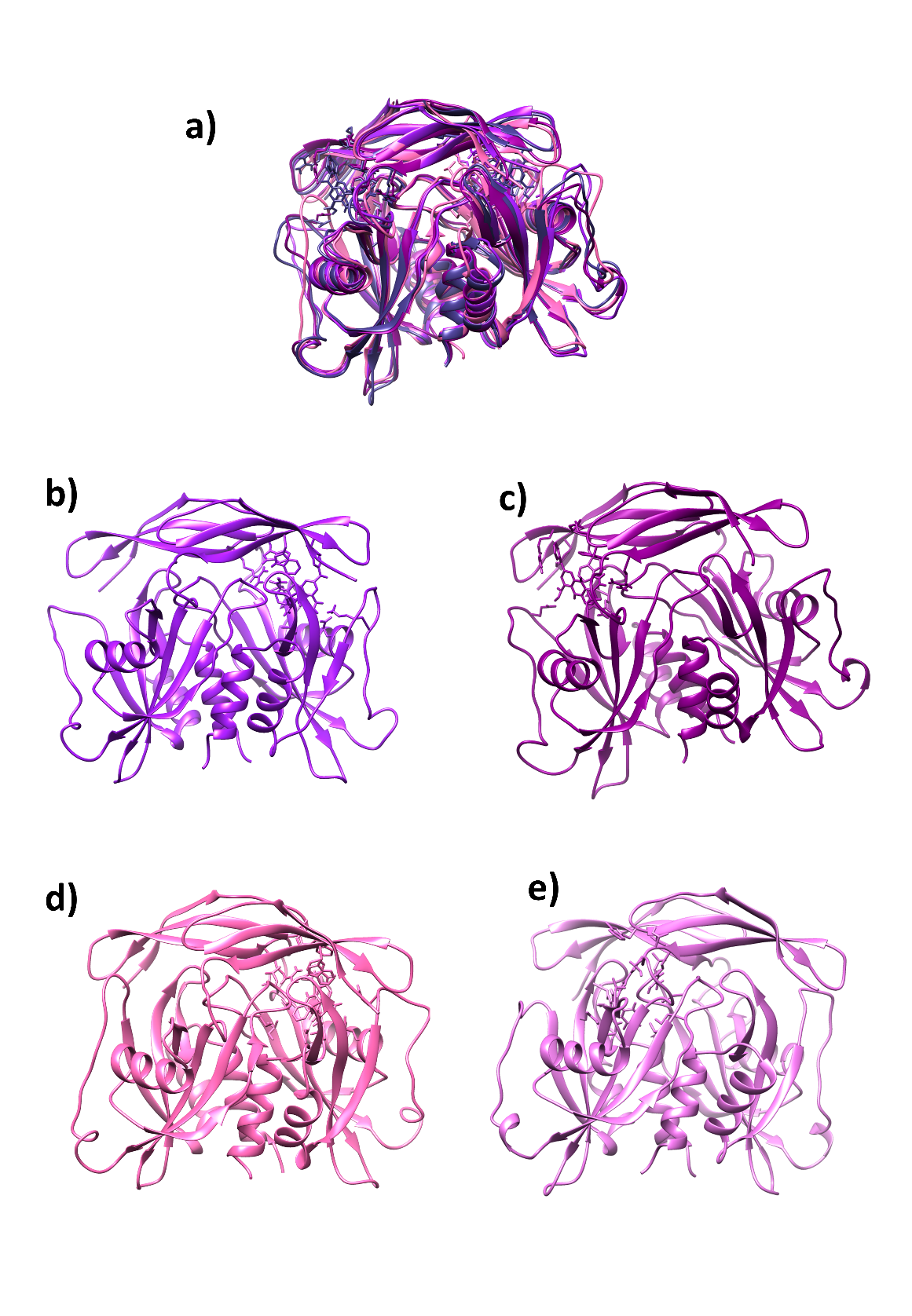


**Figure S4.** (a) Superimposition of the initial pose of the NUDT5-CMNPD19658 complex with the top-four minimum energy poses. (b, c, d, and e) Respective structures with minimum global energy.


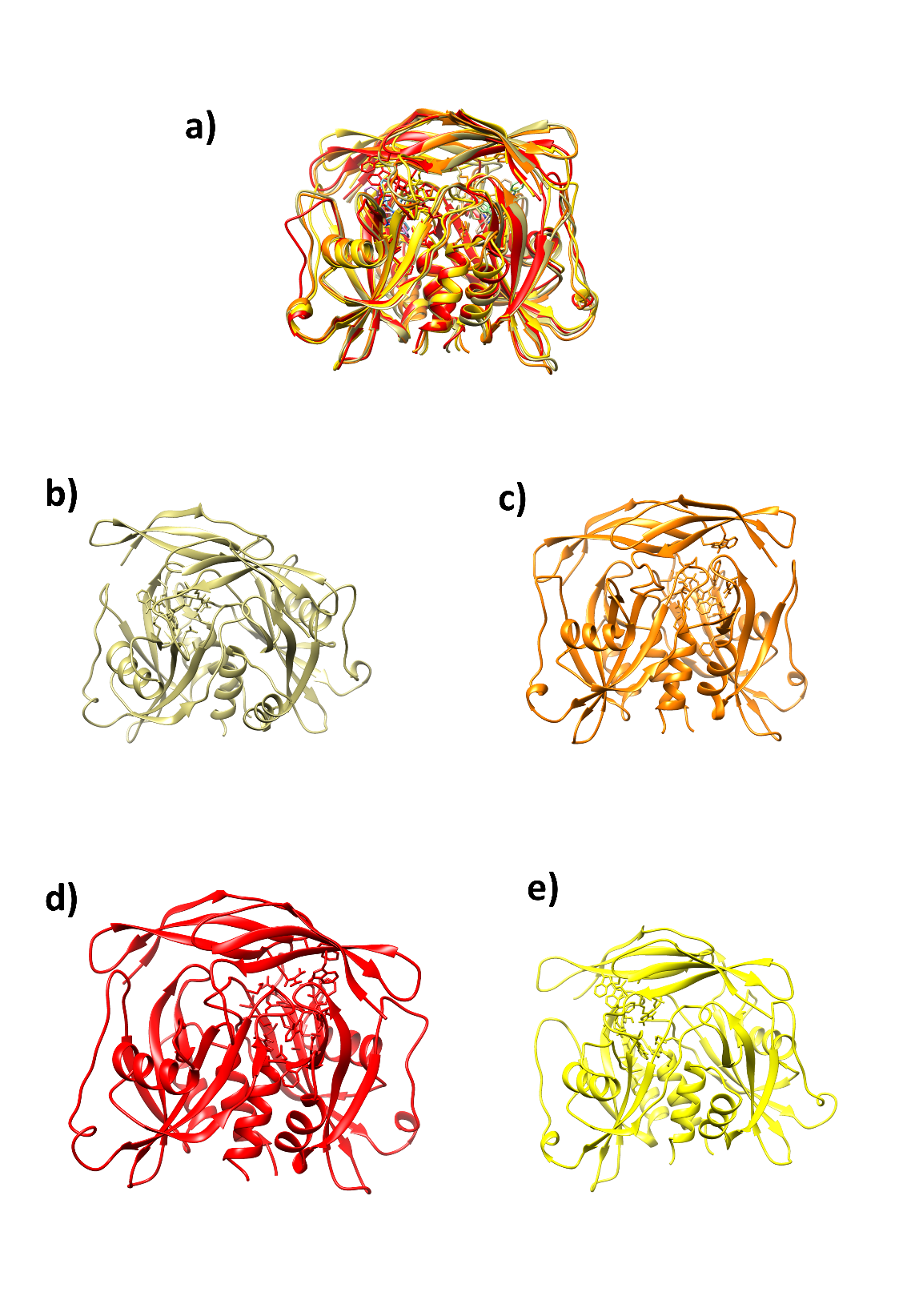


**Figure S5.** (a) Superimposition of the initial pose of the reference complex NUDT5-958 with the top-four minimum-energy poses. (b, c, d, and e) Respective structures with minimum global energy.
